# The Optimal Number of Surveys when Detectability Varies

**DOI:** 10.1371/journal.pone.0115345

**Published:** 2014-12-19

**Authors:** Alana L. Moore, Michael A. McCarthy, Kirsten M. Parris, Joslin L. Moore

**Affiliations:** 1 Laboratoire de modélisation, CNRS-UMR Écologie des Forêts de Guyane, Kourou, French Guiana; 2 School of Botany, The University of Melbourne, Parkville, Victoria, Australia; 3 Australian Research Centre for Urban Ecology, Royal Botanic Gardens Melbourne, Parkville, Victoria, Australia; 4 School of Biological Sciences, Monash University, Melbourne, Victoria, Australia; Institut Maurice-Lamontagne, Canada

## Abstract

The survey of plant and animal populations is central to undertaking field ecology. However, detection is imperfect, so the absence of a species cannot be determined with certainty. Methods developed to account for imperfect detectability during surveys do not yet account for stochastic variation in detectability over time or space. When each survey entails a fixed cost that is not spent searching (e.g., time required to travel to the site), stochastic detection rates result in a trade-off between the number of surveys and the length of each survey when surveying a single site. We present a model that addresses this trade-off and use it to determine the number of surveys that: 1) maximizes the expected probability of detection over the entire survey period; and 2) is most likely to achieve a minimally-acceptable probability of detection. We illustrate the applicability of our approach using three practical examples (minimum survey effort protocols, number of frog surveys per season, and number of quadrats per site to detect a plant species) and test our model's predictions using data from experimental plant surveys. We find that when maximizing the expected probability of detection, the optimal survey design is most sensitive to the coefficient of variation in the rate of detection and the ratio of the search budget to the travel cost. When maximizing the likelihood of achieving a particular probability of detection, the optimal survey design is most sensitive to the required probability of detection, the expected number of detections if the budget were spent only on searching, and the expected number of detections that are missed due to travel costs. We find that accounting for stochasticity in detection rates is likely to be particularly important for designing surveys when detection rates are low. Our model provides a framework to do this.

## Introduction

The probability of detecting a species has important implications for ecological surveys of plants and animals. In an individual survey, detection is often imperfect, that is, the species may be present but remain undetected [1]–[3]. Consequently, the absence of a species cannot be determined with certainty [4], [5]. This needs to be accounted for in order to, for example, derive unbiased estimates of abundance [6], [7]. Imperfect detectability has important consequences in ecology, including for re-visitation studies [8], demographic studies [9], environmental impact assessments [10], species occupancy studies [11], [12] and species distribution models [13].

Detectability is also a key parameter when designing surveys [10], [14]–[17], managing cryptic species [18], [19], designing monitoring programs [20], [21] and managing invasive species [22]–[25]. Several methods have been developed to estimate and account for imperfect detection during ecological surveys [10], [26], [27]. However, to the best of our knowledge, these methods do not account for stochastic variation in detectability, despite such variation being well documented and potentially important [28].

The rate of detection between individual surveys can vary for a range of reasons, including changes in the activity or visibility of the species through time [29], [30], changes in the survey conditions, or variation between observers [31], [32]. Although some of this variability can be predicted in advance (e.g. by considering flowering season), or close to the survey day (e.g. by considering weather conditions), much of it cannot. For example, although we may know the flowering season for a particular plant species, how many plants are flowering on a particular day (and hence the detectability of the species on that day) is not able to be predicted in advance. Even though we do not know the exact detectability on a given day, we can estimate the mean detectability throughout the flowering season and variability about this mean. In the following, when we refer to variability in detection rate, we refer to this kind of stochastic variation in the detection rate.

Consider surveying a single site to determine whether or not a particular species is present. Assuming a particular time budget, observers will aim to spend as much of that time as possible at the survey site. Therefore, if there is no variation in detectability between visits, observers should only visit each site once to minimize fixed costs of travel. However, stochastic variation in detectability between visits results in a trade-off when surveying a single site. The chance of encountering favorable survey conditions during at least one survey increases with the number of surveys at a site. However, extra surveys require extra fixed costs of initiating the surveys (e.g., extra travel time) and shorter individual surveys for the same total time budget. Here we present a model with stochastic detectability that addresses trade-offs between the number of surveys and the length of each survey and examine how this stochasticity impacts on the efficiency of surveys. We analyze the model to determine the number of surveys that is most likely to: 1. maximize the expected probability of detection or 2. achieve a minimally-acceptable probability of detection for given characteristics of the survey.

## Methods

We developed a model of detection at a single site that accounts for stochastic variation in the detection rate between visits. We assumed that detection occurs as a Poisson process and considered both when the detection rate between visits is uncorrelated and correlated through time as two separate cases. We describe the model assuming that we are trying to optimize the number of visits to a single site but note that the derivation is identical when considering the number of sites rather than the number of visits. We used the proposed model to evaluate how stochastic detection rates affect minimum effort requirements for surveying protocols. We also applied the model to two case studies in which we determined the optimal number of visits to a site and the optimal number of quadrats to survey in a region. In the second case study, we had additional data that allowed us to compare the predicted optimal number of quadrats to an empirically-derived estimate to determine the ability of the model to find the optima. Details of these various steps are described below.

Fieldwork associated with the cascade treefrog data was approved by the Australian National University animal experimentation ethics committee, and conducted under the following permits: QDPI permits no. 788, 860 and 919, QNPWS permit no. 2001, QDNR permits no. 1188 and 1301, QDEH permits no. HO/000139/95/SAA and E5/000003/98/SAA, NSW NPWS scientific investigation license no. B1474 and SF NSW permits no. 5267 and 5269.

### The Model

Consider surveying a single site to detect the presence or absence of a particular species. If the species is detected during survey *i* at rate *λ_i_*, then the probability of failing to detect the species in survey *i* when searching for time *t_i_* is exp(−*λ_i_ t_i_*) if encounters occur randomly (as a Poisson process). Assuming that detection rate, *λ_i_*, is independent among surveys (correlations among surveys is considered in S1 Appendix), then the probability of failing to detect the species during *n* surveys is:
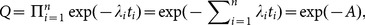
(1)where *A* = 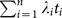 is the expected number of detections for the entire survey period (since *λ_i_* is the mean number of detections per time unit, *λ_i_t_i_* is the expected number of detections in survey *i*). As previously discussed, the rates of detection *λ_i_* might vary from survey period to survey period. If we knew which survey period had the highest rate of detection, the probability of failing to detect the species, *Q*, would be minimized by concentrating effort in the time period for which *λ_i_* is largest. However, we consider the case when the detection rates *λ_i_* are not precisely known prior to surveys and treat them as random variables, with mean *µ* and standard deviation *σ*.

Consider the case where survey effort is divided equally between visits, so there are *n* surveys each of length *t.* Consequently, the mean of *A* is *µ_A_*  =  *nµt*, and the variance is *σ_A_^2^* = *n σ* ^2^ *t* ^2^. We assume that *A* = 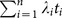 has a log-normal distribution (which will be approximately true if the individual detection rates *λ_i_* have lognormal distributions; Wilkinson's method, e.g. [33] That is, *X* = ln(*A*) is normally distributed with mean *m* = ln(*µ_A_*)–ln(1+*σ_A_* ^2^/*µ_A_* ^2^)/2, and variance *v* = ln(1+*σ_A_* ^2^/*µ_A_* ^2^). Note that while it is useful to define the mean and variance of *A* and *X* in order to derive the objective functions, the final equations are expressed in terms of *µ* and *σ*, the parameters of the detection rate that are estimated.

#### Objective 1: Maximize the expected probability of detection

First, we consider finding the number of surveys that maximizes the expected probability of detection over the entire survey period, or equivalently minimizes the expected probability of failed detection *E*[*Q*]. If *X* is a normal random variable with mean *m* and variance *v*, the cumulative density function (cdf) of *Q*  =  exp(−*A*)  =  exp (−exp[*X*]) is given by,
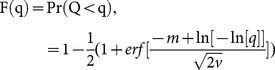
(2)where *m* and *v* are the mean and variance of *X*  =  ln(*A*) as defined above and erf is the normal error function. Let *f*(*q*) denote the probability density function of *q; f*(*q*)* = dF*(*q*)/*dq*. The expected value of *Q* is obtained using the standard formula for expected values (S2 Appendix), 
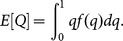



The resulting expression is a decreasing function of both *n* and *t* (S2 Appendix). However, a budget constraint on the total available funding means there is a trade-off between these two aspects of the surveys. Should we increase the number of surveys *n* and decrease the length of each, or vice-versa? We assume that there is a total time budget *B* to survey the site and that each survey has a fixed time *c* that does not contribute to detections (*e.g.*, the time taken to travel to and from the site) and a variable time *t* that does contribute to detections. The expected value of *Q* then needs to be minimized subject to the constraint *B*  =  *n*(*c* + *t*). We set *t*  =  *B*/*n* – *c* and substitute for *t* to get an expression for the expected value of *Q* with only one control variable, *n* (S2 Appendix).

For a Poisson process with detection (or arrival) rate *µ*, the mean time until first detection is 1/*µ*. Since the units of the budget and fixed cost are essentially arbitrary unless related to the detection rate, it is useful to express them in units of mean time until first detection. Consequently, we define the scaled budget and scaled fixed-cost as *B*′ =  *Bµ* and *c*′ =  *cµ* respectively. We find that the resulting expression for the expected value of *Q* depends only on three parameter combinations: *B*′, *c*′ and the coefficient of variation *θ*  =  *σ*/*µ* (see S2 Appendix). The final equation for the expected value of *Q* is:
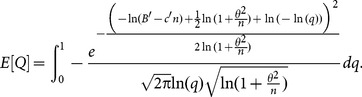
(3)


Note that we only consider integer numbers of surveys when calculating the exact optimal solution where equation 3 is minimized.

#### Analytical approximation for objective 1

While we can use the above expression together with numerical methods to calculate the optimal number of visits for a given set of parameters, it is useful to look for an analytical approximation that may give further insight into key relationships that determine the solution, and that may be more computationally convenient. Using Laplace's method to approximate the integral in Equation 2, we derive the following approximation for the optimal number of surveys that minimizes Equation 2 (see S2 Appendix):
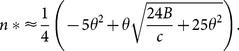
(4)


#### Objective 2: Satisfy a prescribed detection target

The number of surveys, *n*, that maximizes the likelihood that the probability of detection failure is less than *Q_c_* (*i.e*., maximizes the likelihood that the probability of detection is greater than 1 – *Q_c_*) is the solution to the implicit equation (see S2 Appendix)
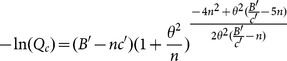
(5)


such that 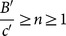.

#### Analytical approximation for objective 2

When *θ* is small, the number of surveys that maximizes the likelihood that the probability of detection failure is less than *Q_c_* can be approximated by (see S2 Appendix)
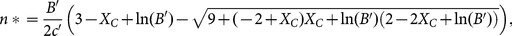
(6)where *X_c_*  =  ln(−ln[*Q_c_*]) is the complementary log-log function of the acceptable probability of detection failure.

### Applications

As elucidated in the introduction, detection probability is a key parameter for many ecological applications. Here we present three simple examples of how the above model may be applied.

1. **Minimum survey effort protocols.** The above model identifies the optimal number of surveys for a given budget but we can also apply the model to identify the budget required to achieve a minimum level of performance. While analytical solutions are not available, numerical approaches can be used to develop minimal effort protocols for both objectives. We illustrate this approach by calculating how the expected probability of detection varies with budget *B* (assuming that the optimal number of surveys is chosen for that budget). Similarly, we determine how Pr(*Q*<*Q*_c_) varies with the budget, again assuming that the optimal number of surveys is chosen. These relationships can then be used to identify the budget required to achieve a sufficient level of performance.
2. **How many surveys?** We applied the analyses to surveys of the cascade treefrog (*Litoria pearsoniana*), using data from searches of 29 stream sites where this species was observed [34]. Each site was a transect 100 m in length along a stream, and was surveyed between 2 and 9 times (mean 3.25 times) between January 1995 and February 1999. The time spent at each site was approximately 1 person hour, with either 2 or 3 people searching the stream and surrounding vegetation for frogs (see Parris 2001 for details of the surveys). Parameter estimates for the model were obtained using Bayesian methods in WinBUGS (OpenBUGS version 3.0.3,[35]). Details of the models and methods used can be found in S3 Appendix.

The parameter estimates were used to predict the average rate of detection and coefficient of variation for sites with 1 detected individual and for sites with 3 detected individuals. These were then used to determine the optimal number of surveys for a range of budgets for the total time, assuming that each site has a fixed time cost of *c* = 1 hour, and survey season was *T* = 3 months (90 nights). These values are consistent with the fixed time costs of travelling to and from each site and the length of the survey seasons reported in Parris (2001).

3. **Testing the model with data: how many quadrats?** The model will always determine the optimal number of surveys if the assumptions are met. The key assumptions are that variation in the summed detection rate (Equation 1) follows a lognormal distribution, and that the mean and standard deviation of that distribution are known. However, both assumptions will be violated in practice; the distribution will not be perfectly lognormal, and the parameters can at best be predicted with error.

We evaluated how well the model predicted the optimal number of surveys for real searches, by using a series of two experiments. In so doing we present another possible application of the model in which we determine the optimal number of sites to survey to detect the presence of a species in a region, or at a smaller scale, the optimal number of quadrats to survey per site. We used an initial search experiment to predict the detection rate of two plant species in a second experiment, with the detection rates predicted to vary among quadrats and searchers. For each of the two species, the predicted mean and standard deviation of the detection rate was used to predict the optimal number of quadrats to survey for each. These predicted optima were compared to the number that would have actually maximized detection of the two species during the second experiment. Details of the experimental plant survey are described by [36], with an overview provided below. While the first experiment used five different species [36], only two of these (*Atriplex semibaccata*, *Lomandra longifolia*) were used in the second experiment, so we report results only for these species.

In the first experiment, nine square (15×15 m) quadrats in an exotic grassland in Royal Park, Melbourne, were planted in 2010 with five species. Thirty, ten, four or two individuals were randomly assigned to each quadrat, and were randomly located within each quadrat. This variation in the density of species among quadrats caused the rate of detection of species to vary among quadrats [36]. Each of 14 observers, who had between 2 and 30 years of plant survey experience, searched the quadrats for 15 minutes and recorded the time to detection of the first and second individual encountered of each species. Average height of the exotic grasses within each quadrat was estimated from 100 point quadrats that were arranged on a square grid at 1.5 m intervals. A failure time model was fitted to the time to detection data from 2010 to estimate the rate of detection of each species within each quadrat by each observer (630 combinations, being 5 species, 14 observers and 9 quadrats). Details of the model can be found in S4 Appendix.

This model and the 2010 data were used to predict detection rates of *Atriplex semibaccata* and *Lomandra longifolia* for each observer and quadrat in 2011, and the average and standard deviation of the detection rates were calculated. From these predictions of detection rate, we predicted the number of quadrats that would maximize the probability of detecting each species in at least one quadrat in 2011 when the search budget was 5, 10 or 15 minutes, and when the time to travel between quadrats was 0.25, 0.5 or 1 minute. This generated nine different values for the optimal number of quadrats, ranging from 1 to 11 quadrats (S4 Appendix).

We also predicted the number of quadrats that optimized the satisficing objective, *i.e.*, that maximized Pr(*Q*<*Q*_c_). We chose *Q*_c_ = exp(−3.0) = 0.05 as the critical probability, which yielded values of between 1 and 16 for the predicted optimal number of quadrats assuming a search budget of 5, 10 or 15 minutes and fixed travel time of 0.25, 0.5 or 1 minute (S4 Appendix).

Search data were collected in 2011 in the same way as in 2010, with the quadrats located in a different section of Royal Park, with the exotic grass being longer on average in the 2011 quadrats. The *L. longifolia* plants used in 2011 were similar in size to those used in 2010, but the *A. semibaccata* plants were noticeably smaller. The different sizes of individuals were not accounted for in the optimization. The only other difference in search protocol in 2011 was that times to detection of all encountered individuals in each quadrat were recorded, and detected individuals were tagged to avoid double counting. All tags were removed before the next observer searched the quadrat.

The number of quadrats that were predicted to be optimal for the 2011 experiment was compared to the empirically-derived optima. The empirically-derived optimal number of quadrats was determined by assuming that quadrat observers would be selected randomly (with replacement) from the different combinations of observers and quadrats. Therefore, the probability of failing to detect a species in a search of length *t* minutes in a single quadrat was “observed” to be *y*/*v*, where *v* is the total number of combinations of observers and quadrats and *y* is the number of those for which the time to first detection was less than *t*. Thus, the probability of failing to detect the species when searching *n* quadrats each for time *t* was (*y*/*v*)*^n^*. Note, *t* is constrained to be *t* = *B*/*n* − *c* so for each combination of *B* and *c*, we found the value of *n* that minimized the probability of failed detection (*y*/*v*)*^n^* (*y* varies with *n*, *B* and *c*). The values for this observed minimum were compared to the predicted minimum determined from our optimal solution that was based on the mean and standard deviation of the detection rate estimated using data from the first experiment (Equation 3).

To test the predictions under the satisficing objective, we note that aiming to achieve a probability of failed detection less than *Q*_c_ is equivalent to achieving an expected number of detections of −ln(*Q*_c_) summed over all quadrats searched (Equation 1). Thus, we determined, the proportion of times that the total expected number of detections for the *n* quadrats would have exceeded −ln(*Q*_c_) (from multiple different random samples of *n* surveys in 2011 and for given values of *B* and *c*). The detection rate for each quadrat and observer in 2011 was calculated as the number of detected individuals divided by the time spent searching the quadrat by the observer. The expected number of detections was calculated as the sum of the detection rate in the sample of *n* quadrats multiplied by the time available to search each quadrat (*B*/*n* – *c*). We then found the value of *n* that maximized the proportion of times that the expected number of detections exceeded −ln(0.05) = 3.0 from 1 million random samples of quadrat searches from the 2011 dataset.

Note that achieving an expected number of detections of −ln(*Q*_c_) is only equivalent to achieving a probability of failed detection less than *Q*_c_ if we assume that detections follow a Poisson process both in the predicted and empirically-derived values (Equation 1). However, the proposed method still tests the other two key model assumptions: that variation in the summed detection rate follows a lognormal distribution, and that the mean and standard deviation of that distribution are known. In contrast, to derive an empirical estimate of the expected probability of failed detection we did not need to assume a model for detections, hence, for the first objective function the assumption that detections follow a Poisson process was also tested.

## Results

Our analysis reveals three variables influence the optimal monitoring design, but their relative influence depends on the choice of objective. The ratio of the budget to the fixed cost per survey was important for both objectives that we examined. When the objective was to maximize the expected probability of detection, variability in the detection rate among surveys, expressed as the coefficient of variation, was important. When considering the second objective, which was to meet a prescribed probability of detection, the coefficient of variation in detection rate was relatively unimportant, while the scaled budget (expected number of detections if the budget were spent entirely on searching and not travelling) became important. These results, and those of the case studies, are described in detail below, with key results summarized in Table 1.

**Table 1:** Summary of key results.

	Objective 1: Maximize the expected probability of detection	Objective 2: Satisfy a prescribed detection target
Key variables	Budget to fixed cost ratio, *B*/*c*	Scaled budget, B′
	Coefficient of variation, *θ* = *σ*/*µ*	Scaled fixed cost, *c*′
		Target probability of failed detection, *Q_c_*
Main results	When the detection rate is highly variable it is optimal to have more, shorter surveys than when it is relatively constant.	The solution is relatively insensitive to changes in the coefficient of variation, *θ*.
		A tougher management objective (lower Q_c_) results in fewer, longer surveys.
	For rare or cryptic species it is optimal to have fewer, longer surveys than for common species.	
	The analytic approximation derived for each objective function performed well.	
Minimum survey effort	When detection rate varies, more effort is required to ensure management objectives are met.	
Treefrog surveys (*B* = 10 hours, *Q_c_* = 0.95)	When the expected abundance is1, 3 surveys are optimal.	When the expected abundance is1, 2 surveys are optimal.
	When the expected abundance is3, 4 surveys are optimal.	When the expected abundance is3, 4 surveys are optimal.
	Correlation between time-steps only affected the solution when the correlation coefficient was quite large.	
Plant surveys *Atriplex semibaccata µ* = 0.55, *σ* = 0.60 *Lomandra longifolia µ* = 0.56, *σ* = 0.64	The predicted optimal number of quadrats ranged between 1 (for both species when budget *B* = 5 and fixed cost *c* = 1) and 11 quadrats (for *L. longifolia* when *B* = 15 and *c* = 0.25).	The predicted optimal number of quadrats ranged between 1 quadrat (for both species when *B* = 5) and 16 quadrats (for both species when *B* = 15 and *c* = 0.25)
(*Q_c_* = 0.95)	The predicted number of quadrats was very close to the empirically-derived optima.	The predicted and observed optimal numbers of quadrats did not correspond as closely but were still strongly correlated.

### General Results

#### Objective 1: Maximize the expected probability of detection

**Figure 1:**
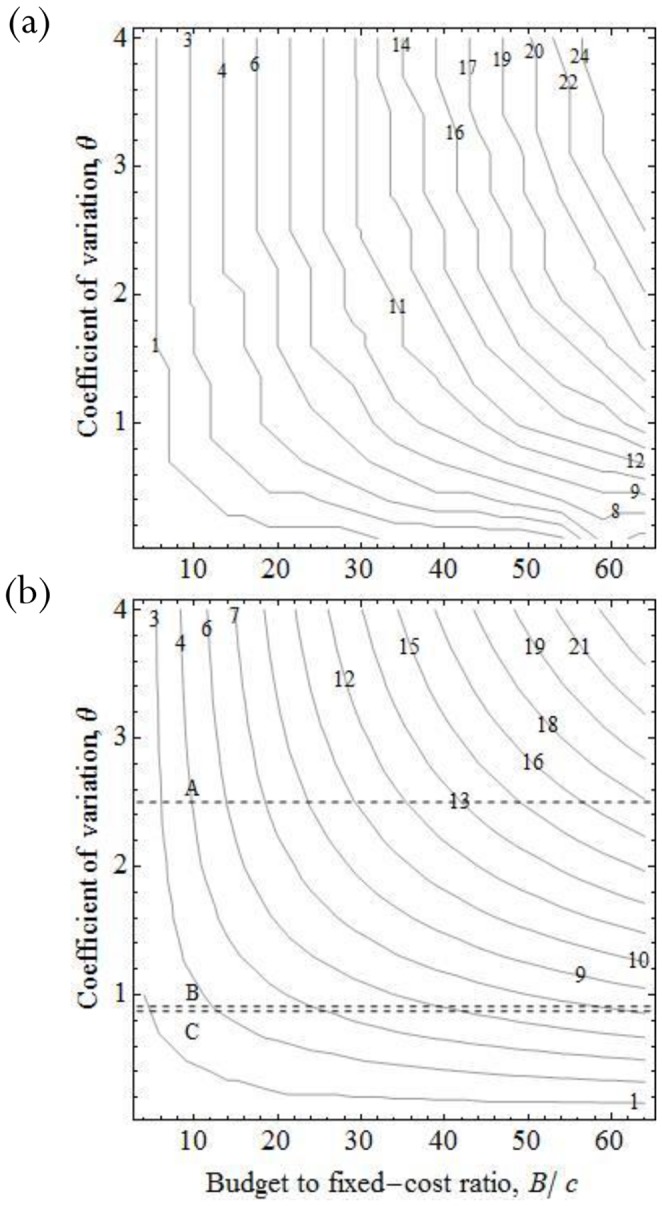
Optimal number of surveys (contours) when maximizing the expected probability of detection as a function of the budget to fixed-cost ratio *B*/*c* ( = *B*′/*c*′) and the coefficient of variation θ. The figures compare the exact solution with *c*′ = 0.5 (a) and approximate solution (b). For the approximate solution, dashed-line A corresponds to *Litoria pearsoniana* (θ = 2.45), dashed-line B corresponds *Atriplex semibaccata* (θ = 0.91) and dashed-line C corresponds *Lomandra longifolia* (θ = 0.87). Note that exact solution depends on the value of *B*, not just the ratio *B*/*c*, hence lines indicating the optimal number of surveys for the case studies are not shown on (a).

As variability in detection increases (*θ* increases), a larger number of surveys per site, each search being of shorter duration, is optimal (Fig. 1a). The optimal number of surveys is also an increasing function of the ratio of budget to fixed cost (Fig. 1a). While the ratio of the budget to fixed cost is influential, the actual values of these two parameters are less so; over more than ten-fold changes in the budget, the optimal number of surveys changes by at most two surveys when the ratio *B*/*c* is held constant (S1 Fig.). Further, only the ratio of these two variables enters into the approximate solution (Equation 3).

While the ratio of the budget to fixed cost is most influential, when the ratio is held constant the exact optimal number of surveys does change with the scaled budget (S1a Fig.); as the scaled budget increases it is optimal to perform more surveys. Consequently, for rare or cryptic species with low detection rates (resulting in a small scaled budget), it is optimal to have fewer, longer surveys than for common species (high mean detection rate, large scaled budget).

Overall, the approximation provided similar results to the full optimization (compare Fig. 1b with 1a). The largest differences between the approximate and exact solution occur when the budget to fixed-cost ratio is large and the coefficient of variation is small (S2 Fig.). However, this had minimal effect on the expected performance because in this region of the parameter space the expected probability of failed detection is very small (S3 Fig.). Consequently, the difference in the value of the objective function is negligible (S3 Fig.). Thus, although the approximation recommends a different number of surveys for some parameter values, the expected performance is nevertheless consistently close to optimal.

#### Objective 2: Satisfy a prescribed detection target

**Figure 2:**
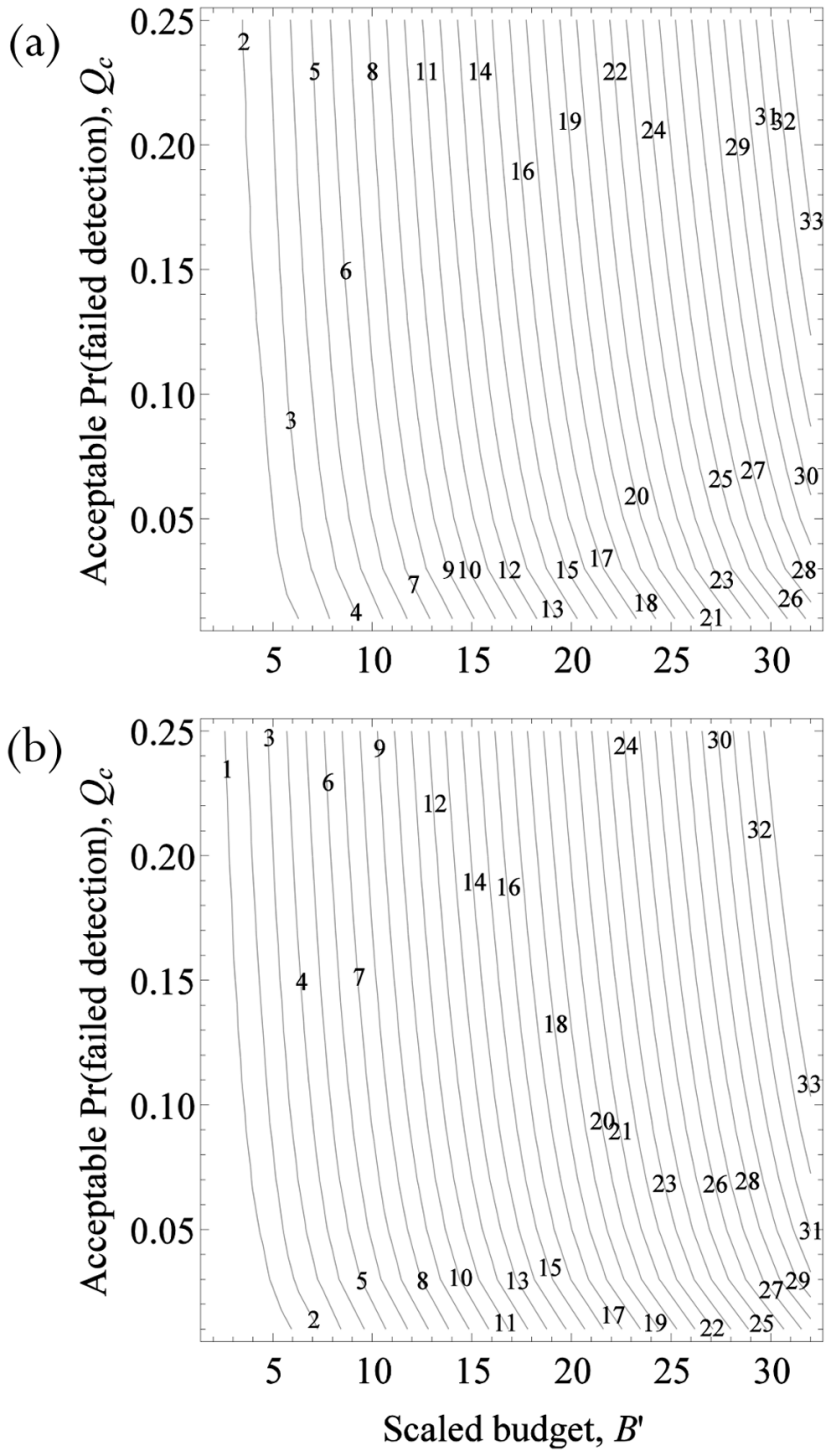
Optimal number of surveys (contours) when maximizing the probability of achieving a prescribed detection rate as a function of the scaled budget *B*′ and the prescribed detection rate *Qc* for the exact solution, with θ = 1.5 and *c*′ = 0.5, (a) and the approximate solution, with *c*′ = 0.5 (b).

When the management aim is to maximize the chance of achieving a minimally-acceptable probability of detection, the optimal solution is largely insensitive to changes in the coefficient of variation *θ* (S4 Fig.). As for the previous objective function, the optimal number of surveys increases with the scaled budget *B*′ (Fig. 2a), and decreases with the scaled fixed-cost *c*′. When maximizing the expected probability of detection, we found that the ratio of budget to fixed-cost that was important, rather than their individual values. However, for the satisficing objective function, the scaled budget is important in its own right; varying the scaled budget while keeping the ratio *B*′/*c*′ ( = B/c) constant has a greater effect on the solution than when maximizing the expected probability of detection (see discussion of approximate solution below).

For this objective function, we have an additional parameter: the prescribed acceptable probability of failed detection *Q_c_*. For a fixed budget and travel cost, a lower prescribed acceptable probability of failed-detection *Q_c_* results in it being optimal to perform fewer, longer surveys (Fig.s 2a).

The approximation derived assuming small *θ* (Equation 5) performs well even when *θ* is reasonably large because the optimal solution is largely insensitive to changes in the coefficient of variation *θ* (S4 & S5 Figs.; for *θ* = 1.5 compare Fig. 2a with 2b). The approximate solution (Equation 6) highlights the substantial influence that the scaled budget *B*′, scaled fixed-cost *c*′ and the management aspiration *Q_c_* have in determining the optimal number of surveys. The scaled fixed-cost *c*′ appears as a scaling factor in the approximate optimal solution, such that if the fixed cost is doubled, the optimal number of surveys will be halved. Consequently, we show results as a function of the scaled budget *B*′ and the management aspiration *Q_c_*, assuming *c*′ = 0.5 for consistency with results for the first objective function.

The approximate solution shows that the optimal number of surveys is also an increasing function of detection rate (higher detection rate implies higher scaled budget) for a given budget and fixed cost. Hence, as for the previous objective function, for rare or cryptic species it is optimal to have fewer, longer surveys than for common species.

### Applications

**Figure 3:**
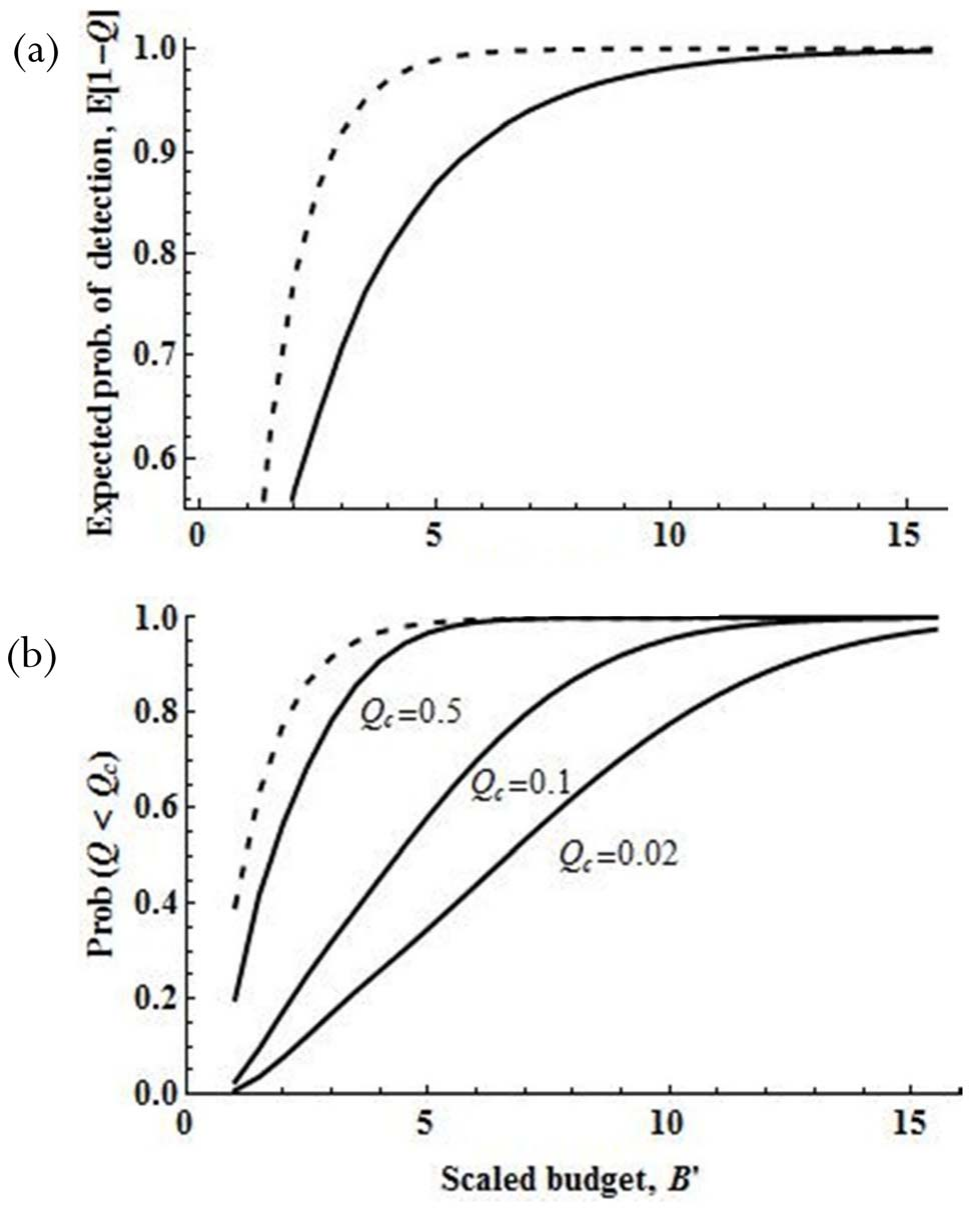
Expected probability of detection (a) as a function of the scaled budget *B*′, with *c*′ = 0.5, when detection rate is assumed to be variable (solid line, θ = 1.5) compared to when it is assumed to be constant (dashed line). Likelihood that the failed-detection probability *Q* is less than the prescribed value *Qc* (b) as a function of the scaled budget *B*′, with θ = 1.5 and *c*′ = 0.5, when detection rate is assumed to be variable (solid lines) compared to when it is assumed to be constant (dashed line).

1. **Minimum survey-effort protocols.** As the variance in the detection rate increases, a greater amount of effort is required to ensure a specified minimum expected probability of detection (Fig. 3a). When the management aim is to maximize the chance that the probability of detection is sufficiently large, rather than consider the effort required to ensure a minimum expected probability of detection, we consider the effort required to ensure that the probability the management goal is achieved, *i.e.,* Pr(*Q*<*Q_c_*) is greater than some minimum level *P_c_* (Fig. 3b). For example, suppose we want to ensure that we detect the species with probability 0.9. If *λ* is constant between visits, this can be achieved in a single visit of at least 3(1/*λ*) time units (Fig. 3b). If *λ* is variable over time, with a coefficient of variation *θ* = 1.5 and scaled fixed-cost *c*′ = 0.5, then with a budget of 3(1/*λ*) time units there is less than 50% chance that the realized detection probability is greater than 0.9 (Fig. 3b). To increase the likelihood that a detection probability of 0.9 is achieved to 90%, the budget needs to be increased to 8.5 (1/*λ*) time units or 12.5 (1/*λ*) time units to increase the likelihood to 98% (Fig. 3b).
2. **How many surveys?** For the case study of *Litoria pearsoniana* the mean detection rate was estimated to be 0.67 detections per hour when the abundance per site was 1 individual, and 2.2 detections per hour when the abundance was 3. The coefficient of variation was 2.5 for both abundance levels. The temporal correlation in detection rate from night to night was estimated to be 0.3, with a wide 95% credible interval of [0.00, 0.97].

**Figure 4:**
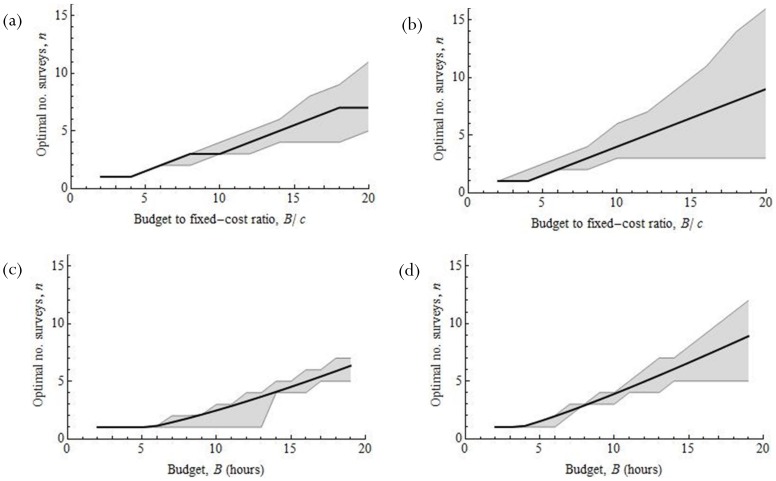
Optimal number of surveys for *Litoria pearsoniana* when the objective is to maximize the expected probability of detection (a & b), and maximize the probability of satisfying a prescribed detection rate of 95% (c & d). Abundance = 1 (µ = 0.67) in (a) & (d), and abundance  = 3 (µ = 2.2) in (b) & (d). The shaded area is the region such that the expected probability of failed detection is no more than 0.01 probability units away from the optimum. The correlation coefficient *r* = 0.3, fixed cost *c* = 1 hour and survey season length *T* = 90 days.

With a budget of 10 hours and an objective to maximize the expected value, it is optimal to perform 3 surveys throughout the season if there is expected to be a single individual (Fig. 4a), or 4 slightly shorter surveys if the expected abundance is 3 (Fig. 4b). The resulting expected probabilities of detection are 0.83 and 0.97 respectively. The correlation between time-steps does not affect the solution unless it is quite large, *r*>∼0.85 for low abundance and *r*>∼0.75 for the higher abundance (S6 Fig.).

The approximation (Equation 3) prescribes 5 surveys for both abundance levels since it is independent of the detection rate (and consequently abundance). This gives expected detection probabilities 0.015 and 0.0012 probability units less than optimum for the lower and higher abundance levels, respectively.

If the objective is to maximize the chance of achieving a detection probability of at least 0.95, it is optimal to perform 2 or 4 surveys throughout the season depending on the expected abundance level (1 or 3 individuals respectively; Fig. 4c & 4d). With such a small budget (10 hours), the correlation between time-steps again does not affect the solution unless the correlation coefficient is high, *r*>∼0.9 (1 individual) and *r*>∼0.85 (3 individuals; S6 Fig.). For both abundance levels and a budget of 10 hours, the approximation (Equation 5) proposes the same number of surveys as calculated using numerical methods.

**Figure 5:**
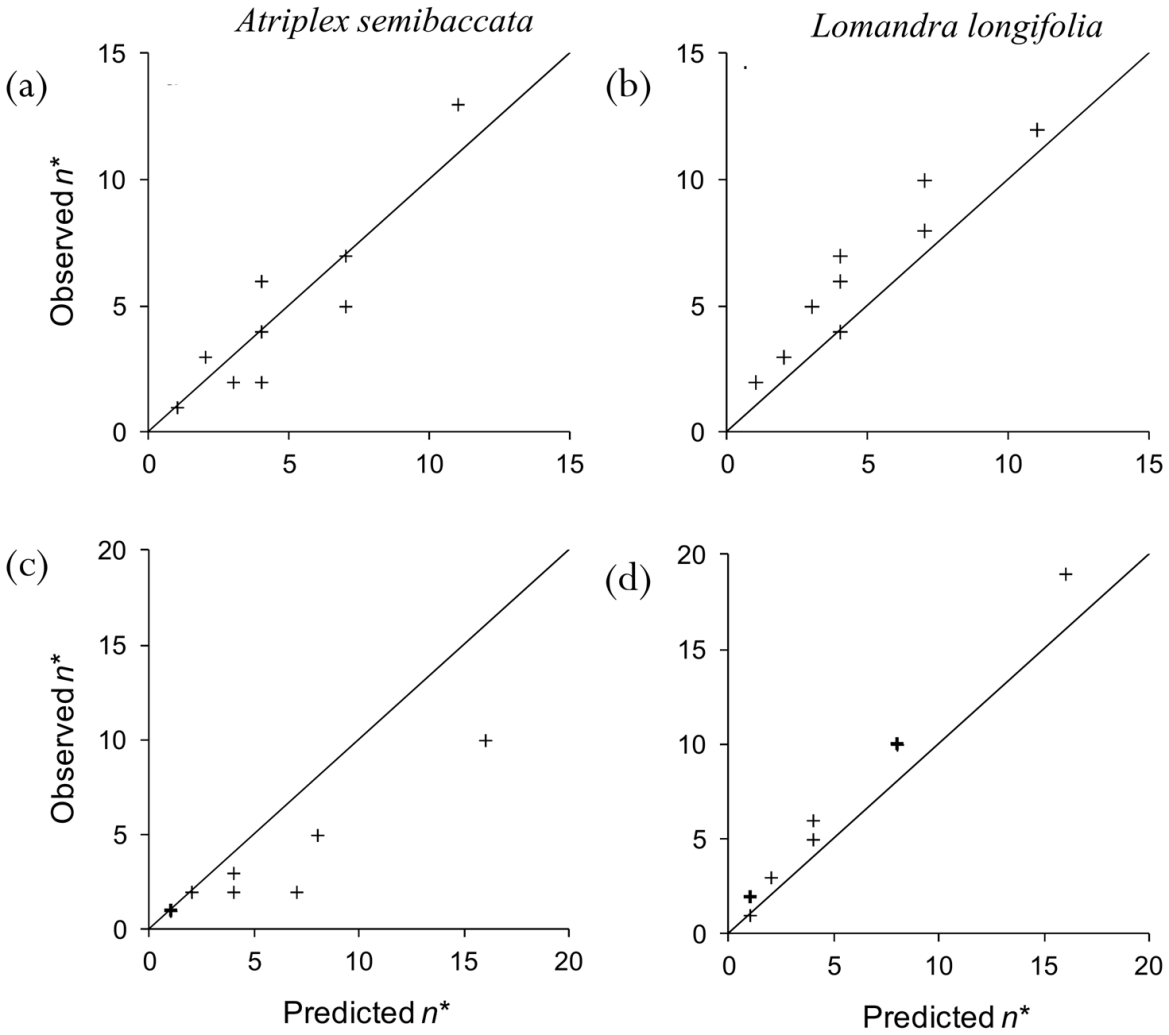
Predicted versus observed optimal number of quadrats to search when: the objective is to maximize the expected probability of detection for *Atriplex semibaccata* (a) and *Lomandra longifolia* (b); the objective is to satisfy a required probability of detection for *Atriplex semibaccata* (c), and *Lomandra longifolia* (d). Multiple values are indicated by the bolder points; three values at the point (1,1) for *Atriplex* (c), and two values at point (1,2) for *Lomandra* (d). Search budget *B* is 5,10 and 15 minutes; travel time between quadrats *c* is 0.25, 0.5 and 1 minute. The diagonal line represents perfect correspondence.

3. **Testing the model with data: how many quadrats?** We tested the predictions of our model using data from a search experiment in 2011. The mean detection rate and standard deviation were estimated to be *µ* = 0.55 (s.d. of posterior  = 0.18) and *σ* = 0.60 for *Atriplex semibaccata*, and *µ* = 0.56 (s.d. of posterior  = 0.17) and *σ* = 0.64 for *Lomandra longifolia* based on detection experiments conducted in 2010. Using these estimates, the optimal number of quadrats to maximize the probability of detecting each species at the site ranged between 1 (for both species when *B* = 5 and *c* = 1) and 11 quadrats (for *L. longifolia* when *B* = 15 and *c* = 0.25) (Fig. 5a, b, see also S4 Appendix: Table 1).

The predicted number of quadrats that maximizes the probability of obtaining at least one detection was very close to the empirically-derived optima (Fig. 5a, b). The relationship is approximately 1∶1 for both species (slope of linear regression is 1.09 (s.e. = 0.18) and 1.04 (s.e. = 0.12) for *Atriplex* and *Lomandra* respectively). For both *Atriplex* and *Lomandra*, the predicted and observed optimal number of quadrats are strongly correlated (*r* = 0.93 and 0.95 respectively for the Pearson product-moment correlation coefficients). This close correspondence occurred despite the detection rates in 2011 differing from those estimated from the 2010 data. For *Lomandra*, the mean detection rate in 2011 was slightly higher than that predicted from the 2010 data (observed rate of 0.61 compared with the prediction of 0.56), while for *Atriplex*, the mean detection rate estimated from data in 2010 was substantially smaller than that observed in 2011 (observed rate of 0.32 compared with the prediction of 0.55). However, for both species, the predicted coefficient of variation in the detection rate was sufficiently close to that observed that the predicted optimal number of quadrats was similar (observed 0.82 for both species, compared with predicted value of 1.09 and 1.14 for *Atriplex* and *Lomandra* respectively).

The optimal number of quadrats predicted to maximize the chance of achieving a detection probability greater than 0.95 ranged between 1 quadrat (for both species when *B* = 5) and 16 quadrats (for both species when *B* = 15 and *c* = 0.25) (Fig. 5c, d). The predicted and observed optimal numbers of quadrats do not correspond quite as closely for this objective (Fig. 5c, d), particularly in the case of *Atriplex* (slope of linear regression is 0.57 (s.e. = 0.07) and 1.164 (s.e. = 0.03) for *Atriplex* and *Lomandra* respectively). The greater difference between predictions and observations for *Atriplex* arises because of the overprediction of the detection rate in 2011 from the 2010 data. Much closer correspondence between the predictions and observations would have been achieved if the mean detection rate of *Atriplex* in 2011 had been predicted more accurately. Nevertheless, for both *Atriplex* and *Lomandra*, the predicted and observed optimal number of quadrats are strongly correlated (*r* = 0.95 and 0.99, respectively). This analysis helps to validate our model for the optimal number of surveys for maximizing the expected probability of detection, and for satisfying a required rate of detection.

## Discussion

The results show that taking account of stochasticity in detection rate is important for designing effective surveys. Further, the chosen objective of the survey influences both the optimal number of visits and the key parameters that determine the optimal solution. For both management objectives and for a range of parameter values, surveying multiple times was more efficient than a single survey (S7 Fig.). However, the value of the objective functions are generally quite robust to the exact number of surveys chosen, in particular when the total budget available is large relative to the mean time until first detection and when the coefficient of variation of the detection rate is small (see Application 2). This suggests that it is important to take stochastic variation into account, but that performance will likely be robust to uncertainty in parameter estimates.

When the objective is to maximize the probability of achieving a prescribed detection probability, the optimal number of surveys is largely independent of the variance in the detection rate, but instead depends on the required detection probability. This is consistent with previous studies that also consider satisficing objective functions (e.g. [37]). Note that although the survey design may not depend on the variance in the detection rate, the value of the objective function (i.e. the probability of achieving the desired detection level) does. Thus, the variance is still important for (a) setting achievable management targets, and (b) setting minimum survey effort levels.

For each objective function we derived an approximate explicit solution for the optimal number of surveys. These explicit solutions are much easier for managers to implement than calculating the solutions numerically, and may be useful for incorporating stochastic detection rates into more complex optimal survey design problems. Note that although the approximate solution when optimizing the expected probability of detection did not approximate the optimal number of surveys for some parameter values, it consistently performed well in terms of the value of the objective function achieved and is consequently still a useful approximation.

Which objective function is most appropriate will depend on management aims. Increasing the expected probability of detection will reduce the number of sites where false absences occur, which might be desirable when many sites are monitored [16], [38]. However, if only a few sites or a single site is monitored, it may be more appropriate to maximize the chance that the detection probability is greater than some specified threshold. For example, when assessing the conservation status of a site prior to development, establishing a species is not present should be demonstrated such that the probability of failed detection is sufficiently low [5], [10], [39].

Determining the minimum effort required to ensure a desired probability of detection is a useful and common application of estimated detection probabilities. Previous studies assume a constant probability of detection; either assuming a constant probability of detection per visit and calculating the minimum number of visits required (e.g. [4], [5]), or determining the minimum required length of a single visit assuming a constant detection rate [10]. Unsurprisingly, when the detection rateis stochastic, more effort is required to ensure management objectives are met. The model presented provides a way to calculate the extra effort required.

Detectability depends on the abundance of the species at the site being surveyed [36]. However, if we are surveying a new site it is unlikely that we will know the abundance in advance. Our results highlight that the expected performance of the objective function is less sensitive to the number of surveys for species with higher detection rates. Therefore, when abundance is unknown it is preferable to design the survey assuming low abundance (i.e. lower detection rate). Similarly, if two species have the same coefficient of variation and it is desirable to survey both species simultaneously, then planning should be based around the rarest or most cryptic species. This holds true for both objective functions.

We tested our modeling approach using an experimental search for plants. Although not extensive, the test gave very encouraging results. For the two species tested, the predicted optimal number of quadrats to search was close to the observed optima. The correspondence was particularly good considering that the empirically-derived estimate would contain some error due to the randomness in the experimental times to detection.

We have applied our model to the case where detection rate varies over time and between locations. This model could be applied to other situations where the detection rate varies in a manner that cannot be predicted *a priori*. For example, the ability of observers to detect species is often quite variable, even when accounting for experience. Consequently, if conducting surveys to determine that a species is absent (*e.g*., absence of pest species for quarantine and trade purposes), is it better to send a single observer to each site or several observers for a shorter amount of time? Given stochastic variation among observers, our approach could determine this optimal number of observers.

In this study we restrict our attention to the case when the mean of the detection rate is assumed to be constant throughout the identified “good” monitoring period. It is also possible that there will be additional structure, for example the mean detection rate may be known to vary cyclicly over the breeding season, reaching a peak at some particular date. This is the subject of ongoing analyses, but we believe the simple case considered here is still applicable to a wide range of scenarios, and is useful when such structure is unknown.

Previous studies that assess optimal survey designs generally assume that the detection probability of a species is constant over time (e.g. [5], [10], [28]). However, in practice this is unlikely to be the case. Here we have shown that survey designs can be made more efficient if variability in detectability is taken into account. We found that accounting for stochastic variation in detection rates is likely to be particularly important when detection rates are low. Further, the effort required to guarantee a particular probability of detection is likely to be underestimated if stochastic detectability is not accounted for in survey designs. Our results have far reaching ramifications due to the the range of disciplines that rely on plant and animal surveys to inform, monitor and evaluate study outcomes. The model and analyses presented is an important theoretical step in optimal survey design as well as being directly applicable to a range of management applications, including environmental impact assessments, species occupancy studies and designing monitoring programs.

## Supporting Information

S1 Fig Effect of scaled budget on the optimal number of surveys for objective 1. For a constant budget to fixed-cost ratio of 10: (a) Scaled budget *B*′ versus the optimal number of surveys *n*, (b) the corresponding optimal expected probability of detection failure. Black and blue lines correspond to a coefficient of variation equal to 0.5 and 3 respectively. The solid-line corresponds to the exact solution and dashed-line to the approximate solution (Note, in figure (b) the black dashed-line is obscured by the solid black-line).
(PDF)

S2 Fig Difference between exact and approximate solution for objective 1. Difference in optimal number of surveys for the approximate and exact solution (approximate minus exact solution), c′ = 0.5.
(PDF)

S3 Fig Value of objective function for objective 1. (a) Expected probability of failed-detection E[*Q*] for exact solution, (b) difference in expected probability of failed-detection E[*Q*] for exact and approximate solutions. *c*′ = 0.5.
(PDF)

S4 Fig Effect of coefficient of variation on the optimal solution for objective 2. (a) Optimal number of surveys using exact solution, (b) optimal number of surveys using approximate solution, (c) value of objective function using exact solution, (d) difference in objective function when using exact and approximate solutions. *c*′ = 0.5, *Q_c_* = 0.05.
(PDF)

S5 Fig Difference between exact and approximate solution for objective 2. Difference between exact and approximate solution as a function of the scaled budget and (a) coefficient of variation (*Q_c_* = 0.05), and (b) detection target (*θ* = 1.5). *c*′ = 0.5.
(PDF)

S6 Fig Correlation coefficient *r* and the optimal number of surveys for *Litoria pearsoniana*. Effect of correlation coefficient *r* on the optimal number of surveys for *Litoria pearsonia* when the objective is to (a)&(b) maximise the expected probability of detection and (c)&(d) maximise the probability of satisfying a prescribed detection target of 95%. (a)&(c): abundance  = 1 (µ = 0.67); (b)&(d) abundance  = 3 (µ = 2.2). Fixed cost *c* = 1 hour and survey season length *T* = 90 days.
(PDF)

S7 Fig Difference between the optimal solution and a single visit. Difference in the value of the objective function between the optimal solution (solid-line) and a single visit (dashed-line) when the objective is to (a) maximise the expected probability of detection and (b) maximise the probability of satisfying a prescribed detection target of 95%, for 3 different values of the coefficient of variation (purple: θ = 0.5, orange: θ = 1, blue: θ = 3). B/c = 15 for both graphs.
(PDF)

S1 Appendix Accounting for temporal correlation.
(PDF)

S2 Appendix Additional model details.
(PDF)

S3 Appendix Additional model details for application 2: how many surveys?
(PDF)

S4 Appendix Additional model details and results for application 3: how many quadrats?
(PDF)

S1 Dataset OpenBugs code and data for application 2 (cascade treefrog surveys).
(RTF)

S2 Dataset OpenBugs code and 2010 data for application 3 (plant surveys).
(RTF)

S3 Dataset 2011 data for application 3 (plant surveys).
(TXT)
