# Supplementary material for: The Optimal Number of Surveys when Detectability Varies
Source: PLoS One. 2014 Dec 19;9(12):e115345. doi: 10.1371/journal.pone.0115345 (PMC4272285; doi:10.1371/journal.pone.0115345)

Figure S1. **Effect of scaled budget on the optimal number of surveys for objective 1.**

For a constant budget to fixed-cost ratio of 10: (a) Scaled budget *B*' versus the optimal number of surveys *n*, (b) the corresponding optimal expected probability of detection failure. Black and blue lines correspond to a coefficient of variation equal to 0.5 and 3 respectively. The solid-line corresponds to the exact solution and dashed-line to the approximate solution (Note, in figure (b) the black dashed-line is obscured by the solid black-line).

(a)

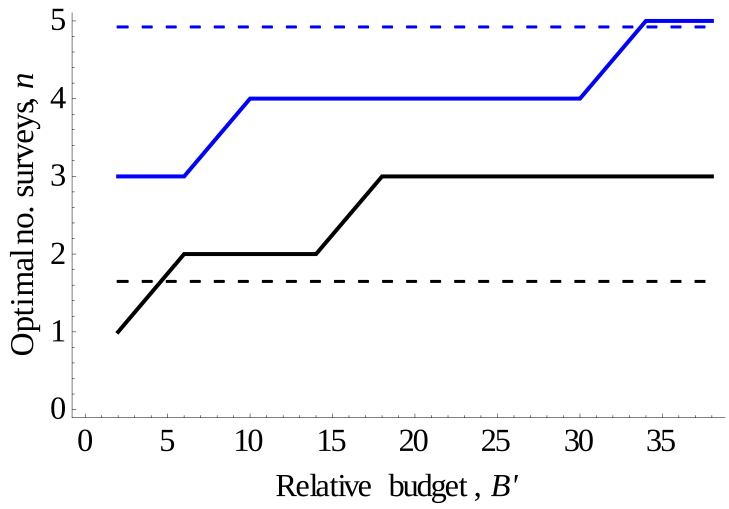

(b)

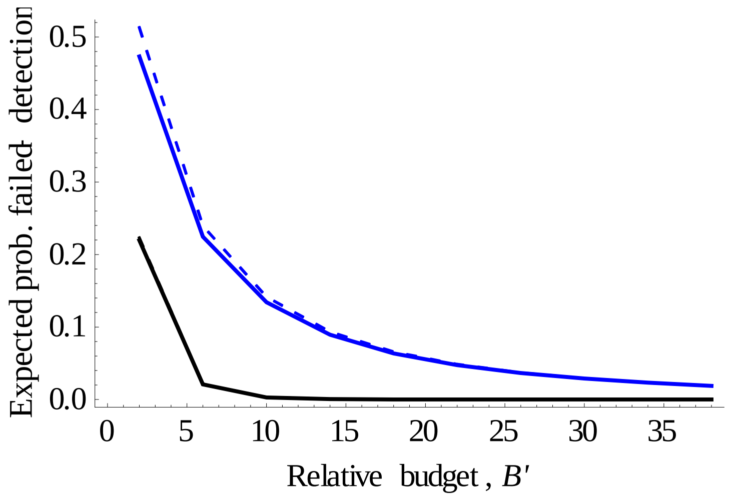

Supplement: S1 Fig — Effect of scaled budget on the optimal number of surveys for objective 1. For a constant budget to fixed-cost ratio of 10: (a) Scaled budget B′ versus the optimal number of surveys n, (b) the corresponding optimal expected probability of detection failure. Black and blue lines correspond to a coefficient of variation equal to 0.5 and 3 respectively. The solid-line corresponds to the exact solution and dashed-line to the approximate solution (Note, in figure (b) the black dashed-line is obscured by the solid black-line). (PDF) [file pone.0115345.s001.pdf]
