# Supplementary material for: The Optimal Number of Surveys when Detectability Varies
Source: PLoS One. 2014 Dec 19;9(12):e115345. doi: 10.1371/journal.pone.0115345 (PMC4272285; doi:10.1371/journal.pone.0115345)

Figure S2. Difference between exact and approximate solution for objective 1. Difference in optimal number of surveys for the approximate and exact solution (approximate minus exact solution), c'=0.5.

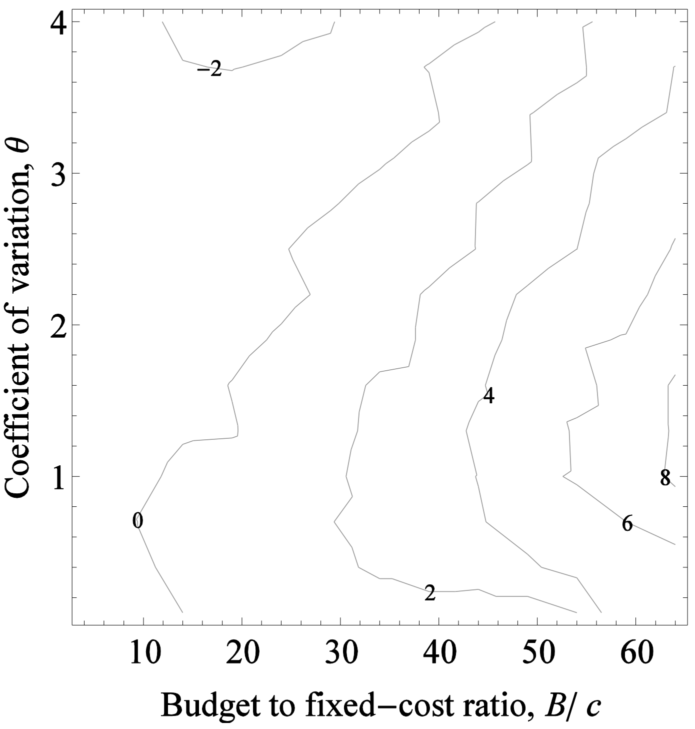

Supplement: S2 Fig — Difference between exact and approximate solution for objective 1. Difference in optimal number of surveys for the approximate and exact solution (approximate minus exact solution), c′ = 0.5. (PDF) [file pone.0115345.s002.pdf]
