# Supplementary material for: The Optimal Number of Surveys when Detectability Varies
Source: PLoS One. 2014 Dec 19;9(12):e115345. doi: 10.1371/journal.pone.0115345 (PMC4272285; doi:10.1371/journal.pone.0115345)

Figure S3. Value of objective function for objective 1. (a) expected probability of failed-detection E[Q] for exact solution, (b) difference in expected probability of failed-detection E[Q] for exact and approximate solutions. c' =0.5.

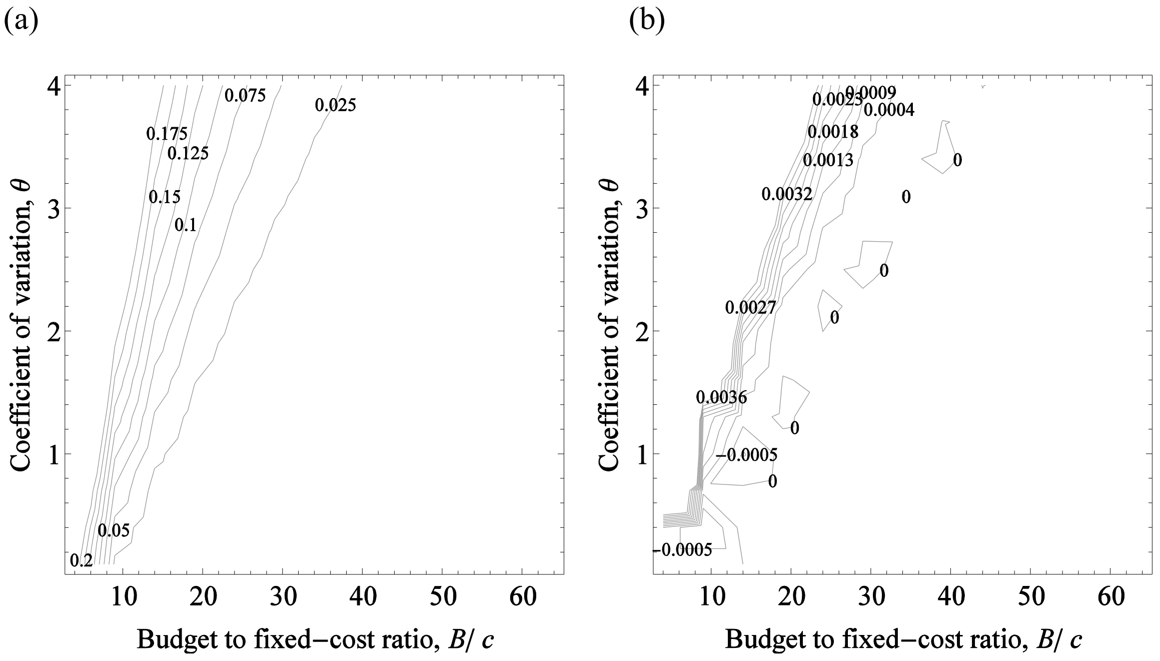

Supplement: S3 Fig — Value of objective function for objective 1. (a) Expected probability of failed-detection E[Q] for exact solution, (b) difference in expected probability of failed-detection E[Q] for exact and approximate solutions. c′ = 0.5. (PDF) [file pone.0115345.s003.pdf]
