# Supplementary material for: The Optimal Number of Surveys when Detectability Varies
Source: PLoS One. 2014 Dec 19;9(12):e115345. doi: 10.1371/journal.pone.0115345 (PMC4272285; doi:10.1371/journal.pone.0115345)

Figure S4. **Effect of coefficient of variation on the optimal solution for objective 2.** (a) Optimal number of surveys using exact solution, (b) optimal number of surveys using approximate solution, (c) value of objective function using exact solution, (d) difference in objective function when using exact and approximate solutions. c' = 0.5, Q_c = 0.05.

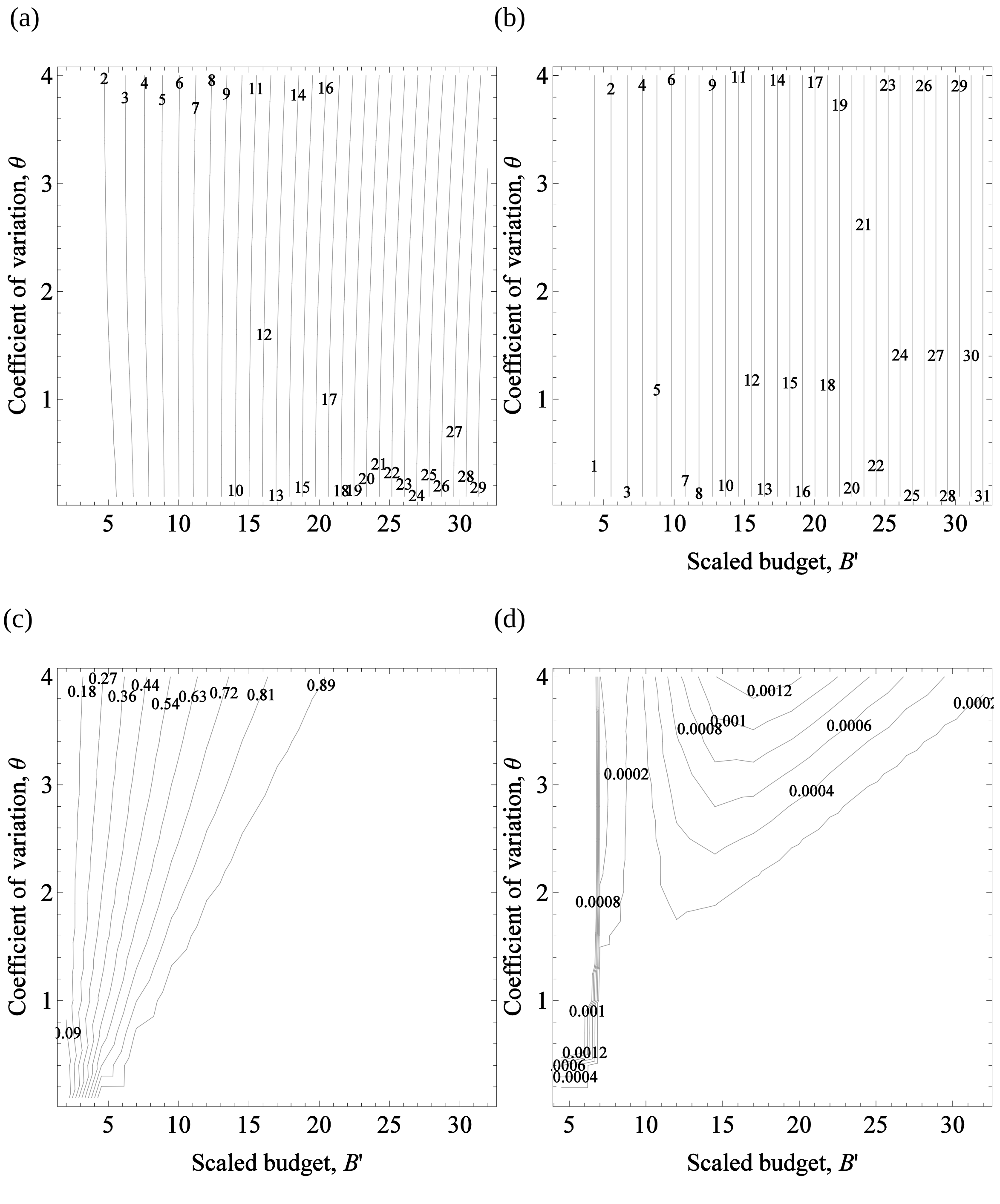

Supplement: S4 Fig — Effect of coefficient of variation on the optimal solution for objective 2. (a) Optimal number of surveys using exact solution, (b) optimal number of surveys using approximate solution, (c) value of objective function using exact solution, (d) difference in objective function when using exact and approximate solutions. c′ = 0.5, Qc = 0.05. (PDF) [file pone.0115345.s004.pdf]
