# Supplementary material for: The Optimal Number of Surveys when Detectability Varies
Source: PLoS One. 2014 Dec 19;9(12):e115345. doi: 10.1371/journal.pone.0115345 (PMC4272285; doi:10.1371/journal.pone.0115345)

Figure S5. Difference between exact and approximate solution for objective 2. Difference between exact and approximate solution as a function of the scaled budget and (a) coefficient of variation (Q_c = 0.05), and (b) detection target (θ = 1.5). c' =0.5.

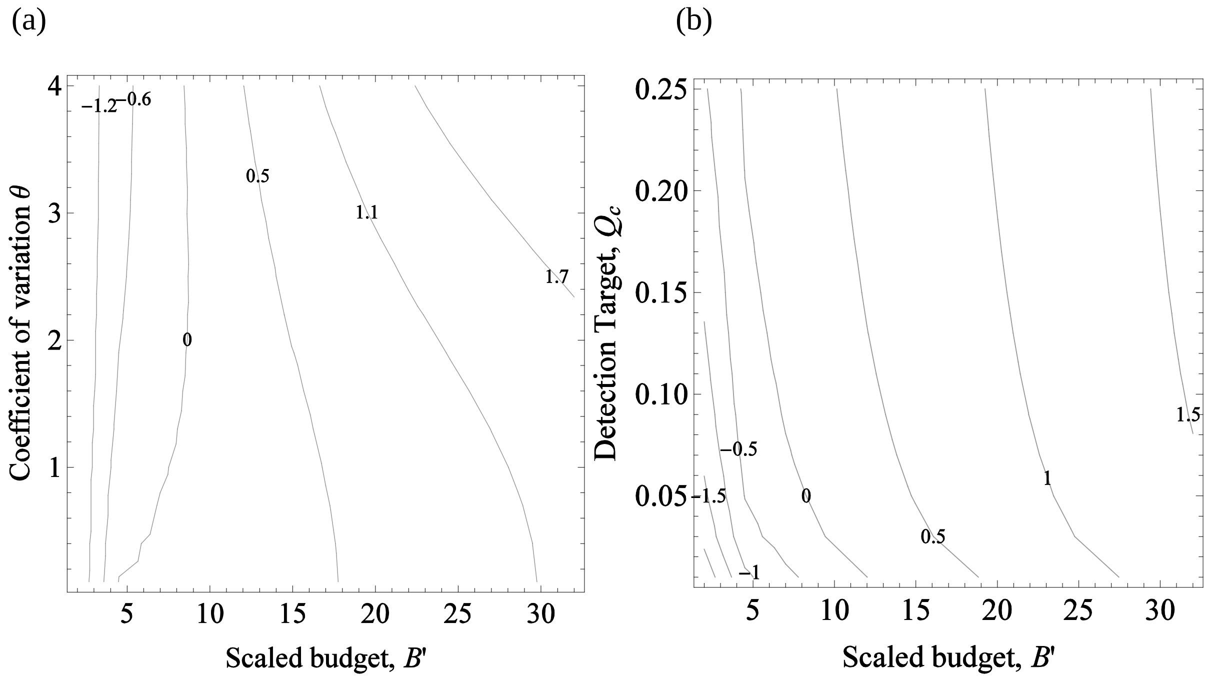

Supplement: S5 Fig — Difference between exact and approximate solution for objective 2. Difference between exact and approximate solution as a function of the scaled budget and (a) coefficient of variation (Qc = 0.05), and (b) detection target (θ = 1.5). c′ = 0.5. (PDF) [file pone.0115345.s005.pdf]
