# Supplementary material for: The Optimal Number of Surveys when Detectability Varies
Source: PLoS One. 2014 Dec 19;9(12):e115345. doi: 10.1371/journal.pone.0115345 (PMC4272285; doi:10.1371/journal.pone.0115345)

Figure S6. **Correlation coefficient *r* and the optimal number of surveys for *Litoria pearsoniana*.** Effect of correlation coefficient *r* on the optimal number of surveys for *Litoria pearsonia* when the objective is to (a)&(b) maximise the expected probability of detection and (c)&(d) maximise the probability of satisfying a prescribed detection target of 95%. (a)&(c): abundance = 1 (μ=0.67); (b)&(d) abundance = 3 (μ=2.2). Fixed cost *c* = 1 hour and survey season length *T* = 90 days.

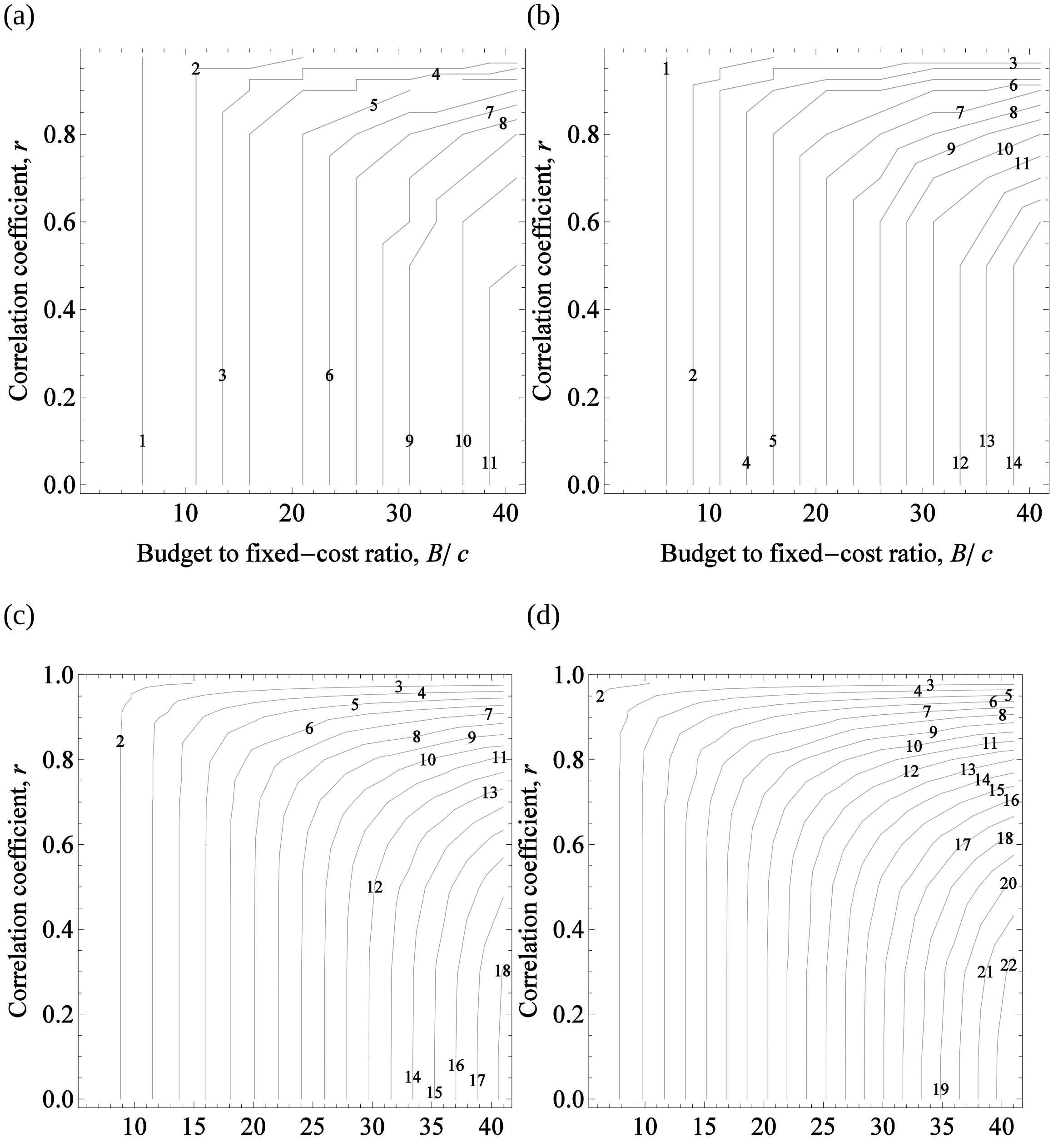

Supplement: S6 Fig — Correlation coefficient r and the optimal number of surveys for Litoria pearsoniana. Effect of correlation coefficient r on the optimal number of surveys for Litoria pearsonia when the objective is to (a)&(b) maximise the expected probability of detection and (c)&(d) maximise the probability of satisfying a prescribed detection target of 95%. (a)&(c): abundance = 1 (µ = 0.67); (b)&(d) abundance = 3 (µ = 2.2). Fixed cost c = 1 hour and survey season length T = 90 days. (PDF) [file pone.0115345.s006.pdf]
