# Supplementary material for: The Optimal Number of Surveys when Detectability Varies
Source: PLoS One. 2014 Dec 19;9(12):e115345. doi: 10.1371/journal.pone.0115345 (PMC4272285; doi:10.1371/journal.pone.0115345)

Figure S7. **Difference between the optimal solution and a single visit.** Difference in the value of the objective function between the optimal solution (solid-line) and a single visit (dashed-line) when the objective is to (a) maximise the expected probability of detection and (b) maximise the probability of satisfying a prescribed detection target of 95%, for 3 different values of the coefficient of variation (purple: θ=0.5, orange: θ=1, blue: θ=3). B/c = 15 for both graphs.

(a)

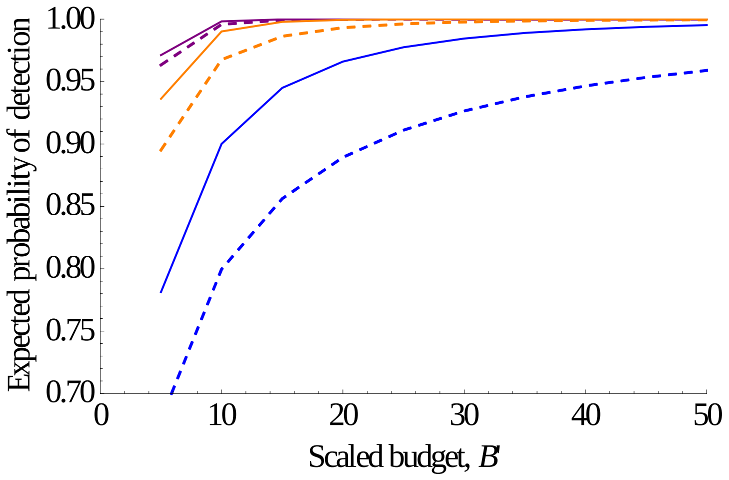

(b)

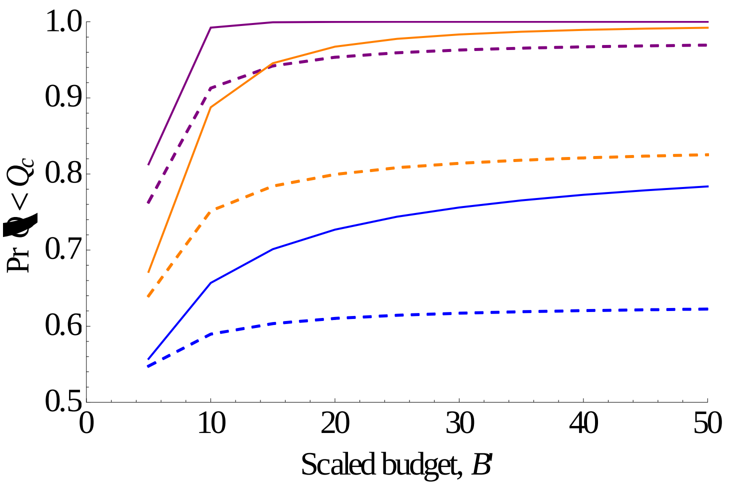

Supplement: S7 Fig — Difference between the optimal solution and a single visit. Difference in the value of the objective function between the optimal solution (solid-line) and a single visit (dashed-line) when the objective is to (a) maximise the expected probability of detection and (b) maximise the probability of satisfying a prescribed detection target of 95%, for 3 different values of the coefficient of variation (purple: θ = 0.5, orange: θ = 1, blue: θ = 3). B/c = 15 for both graphs. (PDF) [file pone.0115345.s007.pdf]
