# Supplementary material for: The Optimal Number of Surveys when Detectability Varies
Source: PLoS One. 2014 Dec 19;9(12):e115345. doi: 10.1371/journal.pone.0115345 (PMC4272285; doi:10.1371/journal.pone.0115345)
Supplement: S1 Appendix — Accounting for temporal correlation. (PDF) [file pone.0115345.s008.pdf]

Appendix S1. Accounting for temporal correlation

The model

The above analysis can be modified to account for correlation in the rate of detection among surveys. If r is the correlation in the detection rates during surveys that are separated in time by one time unit, then the correlation between detection rates that are separated in time by x time units will equal r^x, assuming there is first-order temporal correlation in the detection rates. If it is assumed that there are n surveys each of length t that are evenly-spaced over a survey period of length T, then the correlation between successive surveys is $r^{T/(n-1)}$. In this case, the mean of A is again $n\mu t$, but the variance is $\sigma^2 t^2 (n + 2\sum_{i=1}^{n-1} r^{i\frac{T}{n-1}}[n-i])$.

The expected value of the failed-detection probability is given by,

$$E[Q] = \int_{q=0}^{1} -\frac{e^{-\frac{\left(-2\ln(B'-c'n)+\ln\left(1+\frac{\left(n-nR^{\frac{2}{-1+n}}+2R^{\frac{1}{-1+n}}\left(-1+R^{\frac{n}{-1+n}}\right)\right)\theta^2}{n^2\left(-1+R^{\frac{1}{-1+n}}\right)^2}\right)+2\ln(-\ln(q))\right)^2}{8\ln\left(1+\frac{\left(n-nR^{\frac{2}{-1+n}}+2R^{\frac{1}{-1+n}}\left(-1+R^{\frac{n}{-1+n}}\right)\right)\theta^2}{n^2\left(-1+R^{\frac{1}{-1+n}}\right)^2}\right)}}}{\sqrt{2\pi}\ln(q)\sqrt{\ln\left(1+\frac{\left(n-nR^{\frac{2}{-1+n}}+2R^{\frac{1}{-1+n}}\left(-1+R^{\frac{n}{-1+n}}\right)\right)\theta^2}{n^2\left(-1+R^{\frac{1}{-1+n}}\right)^2}\right)}}dq,$$

where $R = r^T$.

The optimal number of surveys/observers that maximises the probability of realising a failed-detection probability no more than a specified maximum Q_c, is the number that minimises

$$L_R = \frac{-2\ln(B'-c'n)+ln\left(1+\frac{\left(n-nR^{\frac{2}{-1+n}}+2R^{\frac{1}{-1+n}}\left(-1+R^{\frac{n}{-1+n}}\right)\right)\theta^2}{n^2\left(-1+R^{\frac{1}{-1+n}}\right)^2}\right)+2ln(-\ln(Q_c))}{2\sqrt{2}\sqrt{\ln\left(1+\frac{(n-nR^{\frac{2}{-1+n}}+2R^{\frac{1}{-1+n}}(-1+R^{\frac{n}{-1+n}}))\theta^2}{n^2(-1+R^{\frac{1}{-1+n}})^2}\right)}}.$$

Again, we do not have an analytical solution, but we are able calculate the optimal number of visits using numerical methods for a range of parameter values.

Results

The benefit of returning on another occasion (and incurring an extra fixed cost) is reduced in the presence of strong temporal correlation (see Fig. 1). For both objective functions, at the

extreme case of perfect correlation a single visit is optimal. As correlation decreases, the number of visits increases non-linearly until the maximum number of visits (corresponding to no correlation) is reached. However, the maximal number of visits may be reached before the correlation coefficient equals zero. The optimal solution is more sensitive to correlations when the scaled budget is large (see Fig. 1).

(a) (b)

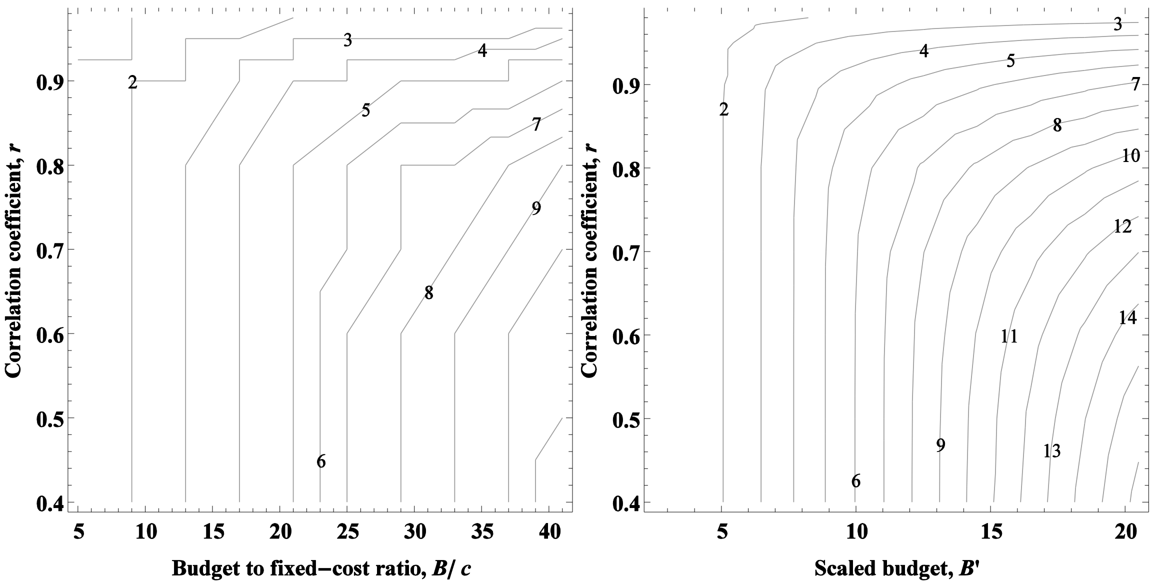

Figure 1. Optimal number of surveys/observers as a function of the correlation coefficient r. (a) Objective is to maximise the expected probability of detection; (b) objective is to maximise the chance of satisfying a prescribed detection target ($Q_c = 0.05$). c=1, v=1.5, μ=0.5.
