# Supplementary material for: The Optimal Number of Surveys when Detectability Varies
Source: PLoS One. 2014 Dec 19;9(12):e115345. doi: 10.1371/journal.pone.0115345 (PMC4272285; doi:10.1371/journal.pone.0115345)
Supplement: S2 Appendix — Additional model details. (PDF) [file pone.0115345.s009.pdf]

Appendix S2. Additional model details

Derivation of objective function 1: Maximise expected probability of detection

As shown in the main text, the cumulative density function (cdf) for the probability of failed-detection, Q, is given by,

$$\mathrm{F(q)} = \mathrm{P}(\mathrm{Q} < q),$$

$$= 1 - \frac{1}{2}\left(1 + \mathrm{erf}\left[\frac{-\mathrm{m}+\ln[-\ln[\mathrm{q}]]}{\sqrt{2}\sqrt{\mathrm{v}}}\right]\right),$$

where m and v are the mean and variance of $X = \ln(A)$ as defined above and erf is the error function. The expected value of Q is obtained using the standard formula:

$$\mathrm{E[Q]} = \int_0^1 \mathrm{q}\,\frac{\mathrm{d\,F(q)}}{\mathrm{dq}}\,\mathrm{dq}$$

$$= \int_0^1 -\frac{e^{-\frac{(-m+\ln(-\ln(q)))^2}{2v}}}{\sqrt{2\pi}\sqrt{v}\ln(q)}\,dq.$$

Substituting for m and v we obtain an expression for the expected value of Q in terms of the mean μ and variance σ^2 of the detection rate λ, number of surveys n and the length of each survey t:

$$\mathrm{E}[Q] = \int_0^1 -\frac{e^{-\frac{\left(-\mathrm{Log}[nt\mu]+\frac{1}{2}\mathrm{Log}[1+\frac{\sigma^2}{n\mu^2}]+\mathrm{Log}[-\mathrm{Log}[p]]\right)^2}{2\mathrm{Log}[1+\frac{\sigma^2}{n\lambda^2}]}}}{\sqrt{2\pi}\mathrm{Log}[p]\sqrt{\mathrm{Log}[1+\frac{\sigma^2}{n\mu^2}]}}\,dp$$

The expected value of Q needs to be minimized subject to the constraint $B = n(c + t)$, where B is the total time budget and c and t are the fixed and variable time associated with each survey respectively. Setting $t = B/n - c$ and substituting this into Equation 2, we get

$$\mathrm{E}[Q] = \int_0^1 -\frac{e^{-\frac{\left(-ln[(-c+\frac{B}{n})n\mu]+\frac{1}{2}\ln[1+\frac{\sigma^2}{n\mu^2}]+\mathrm{Log}[-\mathrm{Log}[q]]\right)^2}{2ln[1+\frac{\sigma^2}{n\mu^2}]}}}{\sqrt{2\pi}\ln[q]\sqrt{\ln[1+\frac{\sigma^2}{n\mu^2}]}}\,dq.$$

Observe that the budget and fixed cost appear in the equation multiplied by the mean detection rate μ. Further, $1/\mu$ is the expected time until the first detection. Consequently, if we express the budget and fixed cost in terms of mean time until first detection, $B' = B\mu$ and $c' = c\mu$, we see that the above expression depends only on three parameter combinations: B', c' and the coefficient of variation $\theta = \sigma/\mu$.

$$\mathrm{E}[Q] = \int_0^1 -\frac{e^{-\frac{\left(-\ln(B'-c'n)+\frac{1}{2}\ln(1+\frac{\theta^2}{n})+\ln(-\ln(q))\right)^2}{2\ln(1+\frac{\theta^2}{n})}}}{\sqrt{2\pi}\ln(q)\sqrt{\ln(1+\frac{\theta^2}{n})}}dq. \quad (1)$$

Analytical approximation

We derive an analytical approximation for $\mathrm{E}[Q]$ using Laplace's approximation:

$$\int f(q)dq \approx \exp\{h(q^*)\}\left(-\frac{2\pi}{h''(q^*)}\right)^{1/2}, \quad (2)$$

where $h(q) = \ln f(q)$ and the global maximum of $h(q)$ occurs at q^*. For the integral in equation A1 we have,

$$h(q) = -\frac{\left(-\ln(B'-c'n)+\frac{1}{2}\ln(1+\frac{\theta^2}{n})+\ln(-\ln(q))\right)^2}{2\ln(1+\frac{\theta^2}{n})} - \ln\left(-\sqrt{2\pi}\ln(q)\sqrt{\ln(1+\frac{\theta^2}{n})}\right),$$

$$h'(q) = -\frac{1}{q\ln(q)} - \frac{-\ln(B'-c'n)+\frac{1}{2}\ln(1+\frac{\theta^2}{n})+\ln(-\ln(q))}{q\ln(q)\sqrt{\ln(1+\frac{\theta^2}{n})}} \text{ and}$$

$$h''(q) = \frac{-2-2(1+\ln(q))\ln(B'-c'n)+3(1+\ln(q))\ln(1+\frac{\theta^2}{n})+2(1+\ln(q))\ln(-\ln(q))}{2q^2\ln(q)^2\ln(1+\frac{\theta^2}{n})}.$$

Setting $h'(q) = 0$ we find the maximum value of h occurs at $q^* = e^{-(B'-c'n)(\frac{n}{n+\theta^2})^{3/2}}$.

Evaluating $h(q)$ and $h''(q)$ at q^*:

$$h(q^*) = \frac{\ln(\frac{n}{n+\theta^2})(2\ln(n(B'-c'n))-2\ln(n+\theta^2)+\ln(2\pi\ln(1+\frac{\theta^2}{n}))}{2\ln(1+\frac{\theta^2}{n})},$$

$$h''(q^*) = -\frac{e^{2(B'-c'n)(\frac{n}{n+\theta^2})^{3/2}}(n+\theta^2)^3}{n^3(B'-c'n)^2\ln(1+\frac{\theta^2}{n})}.$$

Substituting into (A2) and simplifying we get

$$E[Q] \approx e^{-(B'-c'n)(\frac{n}{n+\theta^2})^{3/2}}\sqrt{\frac{n}{n+\theta^2}}.$$

Differentiating with respect to n we find that the optimal number of surveys, n^*, is the solution to the implicit equation

$$B' = \frac{\theta^2(n+\theta^2)^{3/2}+c'n^{5/2}(2n+5\theta^2)}{3n^{3/2}\theta^2},$$

subject to the condition $\frac{B'}{c'} \geq n \geq 1$. We can rewrite this as

$B' = \frac{1}{3}c'n(5 + \frac{2n}{\theta^2}) + \frac{1}{3}(1 + \frac{\theta^2}{n})^{3/2}$.

For large n, the second term, $\frac{1}{3}(1 + \frac{\theta^2}{n})^{3/2}$, tends to be relatively small. By dropping this term and solving for n we get the following approximation for the optimal number of surveys:

$$n^* \approx \frac{1}{4}(-5\theta^2 + \theta\sqrt{\frac{24B'}{c'} + 25\theta^2}) \quad (3)$$

For the range of parameters we considered, the approximate analytical solution was very similar to the number of surveys that maximises the Laplace approximation of the mean detection probability (unpublished data). Consequently, we only show results for the exact solution and the approximate solution given by equation A3.

Derivation of Objective function 2: Satisfy a prescribed detection target

When the probability of failing to detect the species is required to be no more than Q_c (i.e. the probability of detection is required to be at least $1 - Q_c$), from equation A1 we have

$$P(Q < Q_c) = 1 - \frac{1}{2}(1 + \text{erf}[\frac{-m+\ln(-\ln Q_c)}{\sqrt{2}\sqrt{v}}]),$$

where m and v are the mean and variance of $X = \ln A$ respectively and erf is the error function. Since the error function is an increasing function, maximising the probability $Q < Q_c$ is equivalent to minimising

$$L = \frac{-m+\ln(-\ln(Q_c))}{\sqrt{2v}},$$

$$= \frac{-\ln(B'-c'n)+\frac{1}{2}\ln(\frac{n+\theta^2}{n})+\ln(-\ln(Q_c))}{\sqrt{2}\sqrt{\ln(\frac{n+\theta^2}{n})}}.$$

Differentiating and setting the derivative equal to zero, we find that n^* is the solution to the implicit equation:

$$\ln(Q_c) = -(B' - nc')\,(1 + \frac{\theta^2}{n})^{\frac{-4n^2+\theta^2(\frac{B'}{c'}-5n)}{2\theta^2(\frac{B'}{c'}-n)}}, \quad (4)$$

such that $\frac{B'}{c'} \geq n \geq 1$.

Analytical approximation

After observing that the numerical solution is relatively insensitive to coefficient of variation, we derive an analytical approximation for the second objective function by considering the asymptotic solution to equation A4 as the coefficient of variation tends to zero. We have,

$$\lim_{\theta\to 0}\ln(Q_c) = -\lim_{\theta\to 0}(B' - nc')\left(1 + \frac{\theta^2}{n}\right)^{\frac{-4n^2+\theta^2\left(\frac{B'}{c'}-5n\right)}{2\theta^2\left(\frac{B'}{c'}-n\right)}},$$

$= -e^{-\frac{2c'n}{B'-c'n}}(B' - c'n).$

So in the limit as θ goes to zero, n^* is the solution to the implicit equation

$\ln(Q_c) = -e^{-\frac{2c'n}{B'-c'n}}(B' - c'n)$

$$=> \ln(-\ln(Q_c)) = -\frac{2c'n}{B'-c'n} + \ln(B' - c'n) \,,$$

$$= -\frac{2c'n}{B'-c'n} + \ln(B') + \ln(1 - \frac{c'n}{B'}). \qquad (5)$$

For smallish n, $c'n/B' < 1$, hence we can approximate $\ln(1 - c'n/B')$ by $-c'n/B'$. Substituting this back into equation A5 and solving for n, we get the following approximation for the optimal number of surveys:

$$n^* \approx \frac{B'}{2c'}\left(3 - X + \ln(B') - \sqrt{9 + (-2 + X)X + \ln(B')(2 - 2X + \ln(B'))}\right),$$

where $X = \ln(-\ln(Q_c))$.
