# Supplementary material for: The Optimal Number of Surveys when Detectability Varies
Source: PLoS One. 2014 Dec 19;9(12):e115345. doi: 10.1371/journal.pone.0115345 (PMC4272285; doi:10.1371/journal.pone.0115345)
Supplement: S3 Appendix — Additional model details for application 2: how many surveys? (PDF) [file pone.0115345.s010.pdf]

Appendix S3. Additional model details for application 2: how many surveys?

We modelled data on the observation of the frog species during survey *i* as a Bernoulli event with probability q_i

$Y_i \sim \text{dbern}(q_i)$.

$Y_i = 1$ represents the species being detected, $Y_i = 0$ otherwise. The probability of failing to detect the species was assumed to be $q_i = \exp(-\lambda_i\, t_i)$, with $t_i = 1$. The rate of detection on survey *i* (λ_i) was modelled as

$$\ln(\lambda_i) = a + bN_{s_i} + \varepsilon_{d_i},$$

where N_{s_i} is the average logarithm of abundance of the species when detected at site s_i, ε_{d_i} is a random effect for surveys on night d_i, *b* is a regression parameter for the effect of abundance on detection rate, and *a* is an intercept term. The random effects for the night of survey were assumed to be normally distributed with a mean of zero and a standard deviation σ that was estimated. Further, the random effects were assumed to be serially-correlated, with the correlation between rates of detection on successive nights equal to r_N. In this case, the serial correlation in the detection rate λ_i (as opposed to $\ln[\lambda_i]$) is given by $r = (\exp[r_N\, \sigma^2] - 1) / (\exp[\sigma^2] - 1)$ (Johnson and Kotz 1972).

Parameter estimates were obtained using Bayesian methods in WinBUGS (OpenBUGS version 3.0.3, Spiegelhalter et al. 2007) , with vague priors for the parameters so that the estimates were influenced by the data rather than the prior. The prior distributions for fixed effects a and b were normal with mean of zero and standard deviation of 1000. The prior distribution of the standard deviation of the random effect was uniform between 0 and 100, with the upper limit chosen so that it was much larger than the values sampled from the posterior distribution. The prior distribution for the correlation coefficient was uniform between 0 and 1. The parameter estimates were used to predict the average rate of detection for sites with 1 detected individual and for sites with 3 detected individuals. Because the detection rate in the statistical model is assumed to be lognormally distributed, the average detection rate is $\mu = \exp(a + b\ln[N] + \sigma^2/2)$, and the coefficient of variation in detection rate is $\sqrt{(\exp[\sigma^2] - 1)}$.

The posterior distributions of the parameters were estimated from 100,000 samples, after discarding a burn-in of 10,000 samples, which was sufficient to ensure the samples were from

the stationary distribution. Stationarity was assessed by inspecting the sequence of samples from three different sets of initial parameter values.
