# Supplementary material for: The Optimal Number of Surveys when Detectability Varies
Source: PLoS One. 2014 Dec 19;9(12):e115345. doi: 10.1371/journal.pone.0115345 (PMC4272285; doi:10.1371/journal.pone.0115345)
Supplement: S4 Appendix — Additional model details and results for application 3: how many quadrats? (PDF) [file pone.0115345.s011.pdf]

Appendix S4. Additional model details and results for application 3: how many quadrats?

Model details

A failure time model was fitted to the time to detection data from 2010 to estimate the rate of detection of each species within each quadrat by each searcher (630 combinations, being 5 species, 14 searchers and 9 quadrats). The model for the rate of detection of species *i* in quadrat *j* by searcher *k* (r_{ijk}) was:

$$\ln(r_{ijk}) = a_i + b\times\ln(n_{ij}) + c\times s_{ijk} + d\times q_{jk} + e\times h_j + f\times exp_k + g\times day_k + \varepsilon_{ij} + \zeta_{jk},$$

where a_i is the intercept term for species *i*, and *b*, *c*, *d*, *e*, *f* and *g* are regression coefficients for effects of explanatory variables. These explanatory variables are the number of individuals of species *i* in quadrat *j* (n_{ij}), a binary variable indicating whether the individual being detected was the first (s_{ijk} =0) or the second found in the quadrat (s_{ijk} = 1), a binary variable indicating whether the quadrat being searched was the first for that searcher (q_{jk} =1) or not (q_{jk} = 0), and the height of the exotic vegetation in the quadrat (h_j). Finally, ε_{ij} and ζ_{jk} are random effects to account for unmodelled differences among searchers and quadrats for each species. The original analysis (McCarthy et al. 2013) included a fixed effect to accommodate different detection rates among days. However, we wished to predict to novel days, so we excluded the day effect, with variation among days subsumed by extra variation among searchers. The random effects were modelled as being drawn from normal distributions with mean of zero and standard deviations (σ_ε, σ_ζ) that were estimated:

ε_{ik} ~ dnorm(0, σ_ε), and

ζ_{jk} ~ dnorm(0, σ_ζ).

Results

We predicted the optimal number of quadrats for each objective function when the search budget was 5, 10 or 15 minutes, and when the time to travel between quadrats was 0.25, 0.5 or 1 minute. For each objective function, this generated nine different values for the optimal number of quadrats (Table D1).

The predicted optimal number of quadrats to search are very similar for the two species as the estimated mean detectability and standard deviation are similar (μ = 0.55 and σ = 0.60 for

Atriplex semibaccata, and μ = 0.56 and σ = 0.64 for *Lomandra longifolia* based on detection experiments conducted in 2010)(Table 1).

Table 1: Predicted optimal number of quadrats to survey when the search budget is 5, 10 or 15 minutes, and when the time to travel between quadrats is 0.25, 0.5 or 1 minute.

Search budget, *B* (minutes)	Travel time, *c* (minutes)	Predicted optimal number of quadrats, *n**			
		Maximise E[1-Q]		Maximize P(Q < Qc = 0.95)	
		A. semibaccata	*L. longifolia*	*A. semibaccata*	*L. longifolia*
5	0.25	4	4	1	1
5	0.5	2	2	1	1
5	1	1	1	1	1
10	0.25	7	8	7	8
10	0.5	4	5	4	4
10	1	3	3	2	2
15	0.25	11	11	16	16
15	0.5	7	7	8	8
15	1	4	4	4	4

Literature cited

McCarthy, M. A., J. L. Moore, W. K. Morris, K. M. Parris, G. E. Garrard, P. A. Vesk, L. Rumpff, K. M. Giljohann, J. S. Camac, S. S. Bau, T. Friend, B. Harrison, and B. Yue. 2013. The influence of abundance on detectability. Oikos **122**:717-726.
