# Supplementary material for: The Optimal Number of Surveys when Detectability Varies
Source: PLoS One. 2014 Dec 19;9(12):e115345. doi: 10.1371/journal.pone.0115345 (PMC4272285; doi:10.1371/journal.pone.0115345)
Supplement: S1 Dataset — OpenBugs code and data for application 2 (cascade treefrog surveys). (RTF) [file pone.0115345.s012.rtf]

Dataset S1. OpenBugs code and data for application 2 (cascade treefrog surveys)
model
{
  for (i in 1:126)  # for each survey
  {
    log(rate[i]) <- a + b * lnAb[i] + renight[Day[i]]   #  rate of detection a function of abundance (average number seen during surveys) and random effect for night
    p[i] <- 1 - exp(-rate[i])
    Y[i] ~ dbern(p[i])
  }

  dummy <- SiteID[1]   #  site ID is in teh data but not used, so jsut assign it to Dummy so WinBUGS is expecting it.

  # set up the temporally varying random effects so they are correlated.
  # night-to-night correlation is r
  # use standard deviation of 1 for random effect terms initially, then multiply random effect terms by the estiamted standard deviation
  st_norm[1] ~ dnorm(0, 1)   # the first re term is generated independently
  renight[1] <- sdnight * st_norm[1]   # multiply it by the sd
  for (i in 2:1506)  # now for each subsequent night
  {
    st_norm[i] ~ dnorm(0, 1)  # generate an independent normal variate
    co_norm[i] <- r*st_norm[i-1] + sqrt(1- r*r)*st_norm[i]  # use the previous variate and the new one to generate a correlated random variate
    renight[i] <- sdnight * co_norm[i]  # turn that correlated random variate into the random effect by mutliplying it by the sd.
  }

  r ~ dunif(0, 1)  # assume positive temporal correlation
  rln <- (exp(r*sdnight*sdnight) - 1) / (exp(sdnight*sdnight) - 1)  # correlation in log space (correlation in the rates)

  precnight <- 1 / (sdnight * sdnight)   # precision of random effect for nightly variation
  sdnight ~ dunif(0, 100)   # standard deviation of random effect for nightly variation

  a ~ dnorm(0, 1.0E-6)  # vague prior for intercept
  b ~ dnorm(0, 1.0E-6)  # vague prior for effect of abundance

  # Take estimates of a, b, and sdnight, to predict detection rate at sites with 1 or 3 frogs
  # Based on a lognormal distribution: ln(rate) = a + b*ln(Abundance) + 0.5 * sdnight^2
}

Results
		mean	sd	MC_error	val2.5pc	median	val97.5pc	start	sample
	a	-1.379	0.4839	0.01332	-2.423	-1.342	-0.5184	10001	100000
	b	1.096	0.3403	0.009592	0.5149	1.064	1.852	10001	100000
	rln	0.2976	0.3108	0.01321	0.001608	0.1594	0.972	10001	100000
	sdnight	1.395	0.5501	0.01831	0.4614	1.333	2.668	10001	100000


list(a=0, b=0, sdnight=0.1)


Day[]	SiteID[]	Y[]	lnAb[]
1496	50	0	0
1497	55	1	0
1497	63	1	0
1497	65	1	0
796	65	0	0
1133	50	0	0
66	50	0	0
1439	51	0	0
1137	65	1	0
70	50	0	0
73	50	0	0
1506	51	1	0
777	65	0	0
16	50	0	0
20	50	0	0
23	50	0	0
390	55	0	0
392	63	0	0
392	65	1	0
395	50	1	0
397	66	1	0.693147181
1496	66	0	0.693147181
1133	66	0	0.693147181
66	66	0	0.693147181
72	66	0	0.693147181
73	66	0	0.693147181
16	66	0	0.693147181
22	66	0	0.693147181
23	66	0	0.693147181
1481	47	1	0.804718956
1421	47	1	0.804718956
1128	35	1	0.828302217
63	35	1	0.828302217
1437	35	0	0.828302217
67	35	0	0.828302217
71	35	0	0.828302217
13	35	1	0.828302217
17	35	0	0.828302217
21	35	0	0.828302217
386	35	0	0.828302217
1483	116	1	0.879685777
1429	116	1	0.879685777
1430	116	1	0.879685777
1497	64	1	0.895879735
796	64	0	0.895879735
1137	64	0	0.895879735
777	64	0	0.895879735
1481	46	1	0.895879735
1421	46	1	0.895879735
392	64	1	0.895879735
1480	49	0	1.098612289
1420	49	1	1.098612289
1481	45	1	1.354025101
1421	45	1	1.354025101
397	71	0	1.386294361
64	71	0	1.386294361
68	71	0	1.386294361
1136	71	0	1.386294361
1503	71	1	1.386294361
72	71	0	1.386294361
14	71	0	1.386294361
1478	115	1	1.386294361
18	71	0	1.386294361
1419	115	0	1.386294361
22	71	0	1.386294361
398	69	1	1.504585173
1494	69	1	1.504585173
66	69	0	1.504585173
1134	69	1	1.504585173
70	69	1	1.504585173
73	69	1	1.504585173
16	69	1	1.504585173
20	69	0	1.504585173
23	69	1	1.504585173
1480	48	0	1.609437912
1420	48	1	1.609437912
398	70	1	1.675047226
1494	70	1	1.675047226
64	70	1	1.675047226
1134	70	1	1.675047226
68	70	1	1.675047226
70	70	1	1.675047226
14	70	1	1.675047226
18	70	1	1.675047226
20	70	1	1.675047226
1411	117	1	1.700598691
1472	117	1	1.700598691
1466	96	1	1.700598691
1490	96	1	1.700598691
1129	37	1	1.857094498
63	37	1	1.857094498
67	37	1	1.857094498
1438	37	1	1.857094498
13	37	1	1.857094498
74	37	1	1.857094498
17	37	1	1.857094498
388	37	1	1.857094498
24	37	1	1.857094498
1478	124	1	1.868834809
1419	124	1	1.868834809
397	54	1	2.05737778
1496	54	0	2.05737778
1133	54	0	2.05737778
66	54	1	2.05737778
70	54	1	2.05737778
73	54	1	2.05737778
16	54	0	2.05737778
20	54	0	2.05737778
23	54	0	2.05737778
398	60	1	2.124247621
1498	60	1	2.124247621
1498	59	1	2.302585093
390	59	1	2.302585093
397	72	1	2.53227604
64	72	1	2.53227604
68	72	1	2.53227604
1136	72	1	2.53227604
1503	72	1	2.53227604
72	72	1	2.53227604
14	72	0	2.53227604
18	72	0	2.53227604
22	72	1	2.53227604
1434	91	1	2.772588722
401	91	0	2.772588722
1480	44	1	2.943052016
1420	44	1	2.943052016
END
