# Supplementary material for: The Optimal Number of Surveys when Detectability Varies
Source: PLoS One. 2014 Dec 19;9(12):e115345. doi: 10.1371/journal.pone.0115345 (PMC4272285; doi:10.1371/journal.pone.0115345)
Supplement: S2 Dataset — OpenBugs code and 2010 data for application 3 (plant surveys). (RTF) [file pone.0115345.s013.rtf]

Dataset S2. OpenBugs code and 2010 data for application 3 (plant surveys)
model
{
  for (i in 1:630) # for each observation
  {
    T1[i] ~ dexp(r1[i])I(MinT1[i], )
    T2[i] ~ dexp(r2[i])I(MinT2[i], )
    r1[i] <- exp(a[Sp[i]] + b*(log(N[i])-log(10)) + d*First[i] + e*(Height[i]-10) +  f*(log(Experience[i])-log(2.157))  + rep[Person[i], Sp[i]] + req[Quad[i], Sp[i]] )
    r2[i] <- exp(a[Sp[i]] + b*(log(N[i])-log(10)) + c + d*First[i] + e*(Height[i]-10) + f*(log(Experience[i])-log(2.157))  +   rep[Person[i], Sp[i]] + req[Quad[i], Sp[i]] )
  }

  for (i in 1:5)  # a[i] is intercept for species i
  {
    a[i] ~ dnorm(0, 1.0E-6) 
  }
  b ~ dnorm(0, 1.0E-6)  # b is the scaling exponent of abundance
  c ~ dnorm(0, 1.0E-6)  # can include an effect for the second detection time
  d ~ dnorm(0, 1.0E-6)   # can include an effect for the first quadrat surveyed
  e ~ dnorm(0, 1.0E-6)  # e is the effect of the height of vegetation for species i
  f ~ dnorm(0, 1.0E-6)   # effect of experience

  for (i in 1:9)  # for each quadrat
  {
    for (j in 1:5)  # for each species
    {
    req[i, j] ~ dnorm(0, precq)
    }
    avreq[i] <- mean(req[i, ])
  }
  for (i in 1:14)  # for each person
  {
    for (j in 1:5)  # for each species
    {
    rep[i, j] ~ dnorm(0, precp)
    }
    avrep[i] <- mean(rep[i, ])
  }

  sdp ~ dunif(0, 100)
  precp <- 1 / (sdp*sdp)
  sdq ~ dunif(0, 100)
  precq <- 1 / (sdq*sdq)

  avhgt <- mean(Height[])
  avexp <- mean(Experience[])


  # predict to 2011 sites
  for (i in 1:14)
  {
    Predrep[i] ~ dnorm(0, precp)
  }

  for (i in 1:9)
  {
    Predreq[i] ~ dnorm(0, precq)
  }

  # predict to data for 2011
  for (i in 1:81)  # Sp 3 is Atriplex;  Sp 2 for Lomandra
  {
    predrLo[i] <- exp(a[2] + b*(log(PredNLom[i])-log(10)) +  d*PredFirst[1] + e*(PredHeight[i]-10) +  f*(PredExperience[i]-log(2.157))  + Predrep[PredPerson[i]] + Predreq[PredQuad[i]] )
    predrAt[i] <- exp(a[3] + b*(log(PredNAtr[i])-log(10)) +  d*PredFirst[1] + e*(PredHeight[i]-10) +  f*(PredExperience[i]-log(2.157))  + Predrep[PredPerson[i]] + Predreq[PredQuad[i]] )
  }

  avrLo <- mean(predrLo[])
  sdrLo <- sd(predrLo[])

  avrAt <- mean(predrAt[])
  sdrAt <- sd(predrAt[])
 }


# Initial values for some stocahstic nodes
list(a=c(-3,-3,-3,-5,-5), b=1, sdp=0.1, sdq=0.1, sdd=0.1, c=0, d=0, e=0, f=0)


# Data for sites to which predictions will be made in 2011 based on fit to data for 2010 (below)

# PredPerson denotes person identities.
# PredExperinece is log of the number of years of survey experience
# PredQuad is quadrat ID
# PredHeight is height of vegetation in the quadrat (cm)
# PredFirst indicates whether it was the first quadrat surveyed
# PredSp is the species identity
# PredN is the number of individuals of that species in the quadrat
PredPerson[]	PredExperience[]	PredQuad[]	PredHeight[]	PredFirst[]	PredNLom[]	PredNAtr[]
1	1.791759469	1	14.21	1	4	10
1	1.791759469	2	13.635	0	30	30
1	1.791759469	3	20.465	0	10	4
1	1.791759469	4	18.485	0	4	4
1	1.791759469	5	19.935	0	30	30
1	1.791759469	6	24.82	0	10	10
2	1.945910149	1	14.21	0	4	10
2	1.945910149	2	13.635	0	30	30
2	1.945910149	3	20.465	0	10	4
2	1.945910149	4	18.485	0	4	4
2	1.945910149	5	19.935	0	30	30
2	1.945910149	6	24.82	1	10	10
3	2.302585093	1	14.21	0	4	10
3	2.302585093	2	13.635	0	30	30
3	2.302585093	3	20.465	1	10	4
3	2.302585093	4	18.485	0	4	4
3	2.302585093	5	19.935	0	30	30
3	2.302585093	6	24.82	0	10	10
4	2.079441542	1	14.21	1	4	10
4	2.079441542	2	13.635	0	30	30
4	2.079441542	3	20.465	0	10	4
4	2.079441542	4	18.485	0	4	4
4	2.079441542	5	19.935	0	30	30
4	2.079441542	6	24.82	0	10	10
5	0	1	14.21	0	4	10
5	0	2	13.635	0	30	30
5	0	3	20.465	0	10	4
5	0	7	18.295	0	4	30
5	0	8	14.84	0	30	10
5	0	9	16.87	1	10	4
6	0	1	14.21	0	4	10
6	0	2	13.635	0	30	30
6	0	3	20.465	0	10	4
6	0	7	18.295	1	4	30
6	0	8	14.84	0	30	10
6	0	9	16.87	0	10	4
7	1.386294361	1	14.21	1	4	10
7	1.386294361	2	13.635	0	30	30
7	1.386294361	3	20.465	0	10	4
7	1.386294361	7	18.295	0	4	30
7	1.386294361	8	14.84	0	30	10
7	1.386294361	9	16.87	0	10	4
8	2.079441542	4	18.485	0	4	4
8	2.079441542	5	19.935	1	30	30
8	2.079441542	6	24.82	0	10	10
8	2.079441542	7	18.295	0	4	30
8	2.079441542	8	14.84	0	30	10
8	2.079441542	9	16.87	0	10	4
9	2.079441542	4	18.485	0	4	4
9	2.079441542	5	19.935	0	30	30
9	2.079441542	6	24.82	0	10	10
9	2.079441542	7	18.295	0	4	30
9	2.079441542	8	14.84	0	30	10
9	2.079441542	9	16.87	1	10	4
10	2.197224577	1	14.21	0	4	10
10	2.197224577	2	13.635	1	30	30
10	2.197224577	3	20.465	0	10	4
10	2.197224577	7	18.295	0	4	30
10	2.197224577	8	14.84	0	30	10
10	2.197224577	9	16.87	0	10	4
11	3.526360525	1	14.21	0	4	10
11	3.526360525	2	13.635	0	30	30
11	3.526360525	3	20.465	0	10	4
11	3.526360525	4	18.485	0	4	4
11	3.526360525	5	19.935	0	30	30
11	3.526360525	6	24.82	1	10	10
12	2.079441542	4	18.485	0	4	4
12	2.079441542	5	19.935	0	30	30
12	2.079441542	6	24.82	0	10	10
12	2.079441542	7	18.295	0	4	30
12	2.079441542	8	14.84	1	30	10
12	2.079441542	9	16.87	0	10	4
13	2.140066163	1	14.21	0	4	10
13	2.140066163	2	13.635	0	30	30
13	2.140066163	3	20.465	0	10	4
13	2.140066163	7	18.295	1	4	30
13	2.140066163	8	14.84	0	30	10
13	2.140066163	9	16.87	0	10	4
14	2.564949357	7	18.295	0	4	30
14	2.564949357	8	14.84	0	30	10
14	2.564949357	9	16.87	1	10	4
END						


# Data for 2010 surveys

# Person denotes person identities.
# Experinece is the number of years of survey experience
# Quad is quadrat ID
# Height is height of vegetation in the quadrat (cm)
# First indicates whether it was the first quadrat surveyed
# Sp is the species identity (1 is Dianella; 2 is Lomandra; 3 is Atriplex; 4 is Einadia; 5 is Themeda)
# N is the number of individuals of that species in the quadrat
# T1 is the time taken to find the first individual of the species in the quadrat (minutes)
# T2 is the time taken (since finding the first) to find the second individual of the species in the quadrat (minutes)
# NA values for T1 or T2 indicate the species was not found a first or second time
# MinT1 is the censored search time if the first individual of the species was not detected; if found MinT1 is zero
# MinT2 is the censored search time if the second individual of the species was not detected (time since finding the first until the end of searching); if found MinT2 is zero


Person[]	Experience[]	Quad[]	Height[]	First[]	Sp[]	N[]	T1[]	T2[]	MinT1[]	MinT2[]
1	4	1	18.94	0	1	2	9.716666667	2.316666667	0	0
1	4	2	10.18	0	1	10	0.116666667	3.966666667	0	0
1	4	3	7.84	0	1	2	8.516666667	4.8	0	0
1	4	4	16.61	0	1	30	3.366666667	0.55	0	0
1	4	5	8.88	0	1	10	2.866666667	2.15	0	0
1	4	6	7.54	1	1	2	2.366666667	6.516666667	0	0
1	4	7	6.53	0	1	30	0.166666667	0.25	0	0
1	4	8	8.56	0	1	2	10.78333333	NA	0	4.216666667
1	4	9	14.67	0	1	10	0.65	2.016666667	0	0
2	2	1	18.94	0	1	2	NA	NA	15	0
2	2	2	10.18	0	1	10	2.933333333	2.2	0	0
2	2	3	7.84	1	1	2	6.3	NA	0	8.7
2	2	4	16.61	0	1	30	0.816666667	1.2	0	0
2	2	5	8.88	0	1	10	1.5	2.366666667	0	0
2	2	6	7.54	0	1	2	13.43333333	NA	0	1.566666667
2	2	7	6.53	0	1	30	1.083333333	1.466666667	0	0
2	2	8	8.56	0	1	2	NA	NA	15	0
2	2	9	14.67	0	1	10	1.216666667	3.416666667	0	0
3	11	1	18.94	1	1	2	14.36666667	NA	0	0.633333333
3	11	2	10.18	0	1	10	1.85	5.083333333	0	0
3	11	3	7.84	0	1	2	6.333333333	7.15	0	0
3	11	4	16.61	0	1	30	0.283333333	0.016666667	0	0
3	11	5	8.88	0	1	10	1.6	0.133333333	0	0
3	11	6	7.54	0	1	2	0.766666667	5.033333333	0	0
3	11	7	6.53	0	1	30	0.05	0.05	0	0
3	11	8	8.56	0	1	2	0.166666667	NA	0	14.83333333
3	11	9	14.67	0	1	10	0.283333333	0.166666667	0	0
4	24	1	18.94	0	1	2	10.6	NA	0	12.4
4	24	2	10.18	0	1	10	0.433333333	0.616666667	0	0
4	24	3	7.84	0	1	2	1.966666667	1.2	0	0
4	24	4	16.61	0	1	30	0.216666667	0	0	0
4	24	5	8.88	0	1	10	0.3	0.183333333	0	0
4	24	6	7.54	0	1	2	3.95	0.633333333	0	0
4	24	7	6.53	0	1	30	0.416666667	0.383333333	0	0
4	24	8	8.56	0	1	2	7.266666667	NA	0	7.733333333
4	24	9	14.67	1	1	10	1.983333333	1.15	0	0
5	17	1	18.94	0	1	2	NA	NA	15	0
5	17	2	10.18	0	1	10	0.566666667	1.533333333	0	0
5	17	3	7.84	0	1	2	2.9	2.05	0	0
5	17	4	16.61	0	1	30	0.2	1.416666667	0	0
5	17	5	8.88	0	1	10	3.033333333	0.016666667	0	0
5	17	6	7.54	0	1	2	4.166666667	1.033333333	0	0
5	17	7	6.53	1	1	30	0.133333333	0.016666667	0	0
5	17	8	8.56	0	1	2	2.866666667	4.866666667	0	0
5	17	9	14.67	0	1	10	0.7	3.266666667	0	0
6	21	1	18.94	0	1	2	NA	NA	15	0
6	21	2	10.18	1	1	10	0.816666667	4.883333333	0	0
6	21	3	7.84	0	1	2	0.683333333	NA	0	14.31666667
6	21	4	16.61	0	1	30	0.216666667	0.916666667	0	0
6	21	5	8.88	0	1	10	2	5.133333333	0	0
6	21	6	7.54	0	1	2	1.983333333	0.316666667	0	0
6	21	7	6.53	0	1	30	2.25	0.016666667	0	0
6	21	8	8.56	0	1	2	NA	NA	15	0
6	21	9	14.67	0	1	10	5.816666667	0.066666667	0	0
7	5	1	18.94	0	1	2	NA	NA	15	0
7	5	2	10.18	0	1	10	1.233333333	0.5	0	0
7	5	3	7.84	0	1	2	0.95	7.133333333	0	0
7	5	4	16.61	0	1	30	0.383333333	0.916666667	0	0
7	5	5	8.88	1	1	10	0.35	0.7	0	0
7	5	6	7.54	0	1	2	2.55	NA	0	12.45
7	5	7	6.53	0	1	30	0.05	0.016666667	0	0
7	5	8	8.56	0	1	2	NA	NA	15	0
7	5	9	14.67	0	1	10	1.016666667	1.083333333	0	0
8	8	1	18.94	0	1	2	5.966666667	NA	0	9.033333333
8	8	2	10.18	0	1	10	6	5.333333333	0	0
8	8	3	7.84	1	1	2	10.68333333	NA	0	4.316666667
8	8	4	16.61	0	1	30	2.416666667	0.266666667	0	0
8	8	5	8.88	0	1	10	0.983333333	6.85	0	0
8	8	6	7.54	0	1	2	13.28333333	0.05	0	0
8	8	7	6.53	0	1	30	0.016666667	0.016666667	0	0
8	8	8	8.56	0	1	2	5.6	1.916666667	0	0
8	8	9	14.67	0	1	10	1.033333333	3.116666667	0	0
9	3	1	18.94	0	1	2	13.26666667	NA	0	1.733333333
9	3	2	10.18	0	1	10	0.55	0.133333333	0	0
9	3	3	7.84	0	1	2	1.166666667	2.683333333	0	0
9	3	4	16.61	0	1	30	0.4	1.416666667	0	0
9	3	5	8.88	0	1	10	0.716666667	1.95	0	0
9	3	6	7.54	0	1	2	5.416666667	1.366666667	0	0
9	3	7	6.53	0	1	30	0.1	0.083333333	0	0
9	3	8	8.56	0	1	2	2.033333333	2.983333333	0	0
9	3	9	14.67	1	1	10	0.616666667	0.616666667	0	0
10	3	1	18.94	0	1	2	0.833333333	NA	0	14.16666667
10	3	2	10.18	0	1	10	0.15	0.283333333	0	0
10	3	3	7.84	0	1	2	1.65	5.483333333	0	0
10	3	4	16.61	0	1	30	0.583333333	3.816666667	0	0
10	3	5	8.88	0	1	10	0.8	2.95	0	0
10	3	6	7.54	0	1	2	5.833333333	NA	0	9.166666667
10	3	7	6.53	0	1	30	0.2	0	0	0
10	3	8	8.56	1	1	2	0.716666667	10.05	0	0
10	3	9	14.67	0	1	10	1.75	1.316666667	0	0
11	10	1	18.94	0	1	2	13.85	NA	0	1.15
11	10	2	10.18	0	1	10	4.516666667	4.933333333	0	0
11	10	3	7.84	0	1	2	3.3	1.9	0	0
11	10	4	16.61	0	1	30	0.233333333	0.266666667	0	0
11	10	5	8.88	0	1	10	0.666666667	8.566666667	0	0
11	10	6	7.54	0	1	2	9.783333333	0.983333333	0	0
11	10	7	6.53	1	1	30	0.25	0.116666667	0	0
11	10	8	8.56	0	1	2	7.75	1.05	0	0
11	10	9	14.67	0	1	10	0.533333333	1.9	0	0
12	16	1	18.94	0	1	2	4.916666667	NA	0	10.08333333
12	16	2	10.18	1	1	10	0.483333333	0.583333333	0	0
12	16	3	7.84	0	1	2	1.433333333	9.016666667	0	0
12	16	4	16.61	0	1	30	0.066666667	0.433333333	0	0
12	16	5	8.88	0	1	10	1.45	1.15	0	0
12	16	6	7.54	0	1	2	0.316666667	2.8	0	0
12	16	7	6.53	0	1	30	1.283333333	0.05	0	0
12	16	8	8.56	0	1	2	3.683333333	1.183333333	0	0
12	16	9	14.67	0	1	10	1.05	0.783333333	0	0
13	30	1	18.94	0	1	2	3.05	NA	0	11.95
13	30	2	10.18	0	1	10	0.966666667	5.066666667	0	0
13	30	3	7.84	0	1	2	8.266666667	1.283333333	0	0
13	30	4	16.61	0	1	30	1.05	0.066666667	0	0
13	30	5	8.88	1	1	10	2.816666667	3.033333333	0	0
13	30	6	7.54	0	1	2	5.466666667	1.116666667	0	0
13	30	7	6.53	0	1	30	0.216666667	0.183333333	0	0
13	30	8	8.56	0	1	2	7.733333333	6.366666667	0	0
13	30	9	14.67	0	1	10	0.05	0.416666667	0	0
14	10	1	18.94	0	1	2	10.48333333	NA	0	4.516666667
14	10	2	10.18	0	1	10	0.333333333	0.083333333	0	0
14	10	3	7.84	1	1	2	1.333333333	6.5	0	0
14	10	4	16.61	0	1	30	0.066666667	0.016666667	0	0
14	10	5	8.88	0	1	10	0.25	0.066666667	0	0
14	10	6	7.54	0	1	2	1.5	1.85	0	0
14	10	7	6.53	0	1	30	0.016666667	0	0	0
14	10	8	8.56	0	1	2	0.816666667	4.05	0	0
14	10	9	14.67	0	1	10	3.783333333	0.883333333	0	0
1	4	1	18.94	0	2	2	2.716666667	11.86666667	0	0
1	4	2	10.18	0	2	10	2.633333333	3.183333333	0	0
1	4	3	7.84	0	2	2	7.816666667	2.366666667	0	0
1	4	4	16.61	0	2	4	12.18333333	NA	0	2.816666667
1	4	5	8.88	0	2	30	0.1	0.066666667	0	0
1	4	6	7.54	1	2	10	10.43333333	0.5	0	0
1	4	7	6.53	0	2	30	0.666666667	0.466666667	0	0
1	4	8	8.56	0	2	2	8.816666667	5.633333333	0	0
1	4	9	14.67	0	2	10	1.15	0	0	0
2	2	1	18.94	0	2	2	0.966666667	11.08333333	0	0
2	2	2	10.18	0	2	10	1.716666667	0.566666667	0	0
2	2	3	7.84	1	2	2	5.983333333	1.466666667	0	0
2	2	4	16.61	0	2	4	NA	NA	15	0
2	2	5	8.88	0	2	30	0.15	1.133333333	0	0
2	2	6	7.54	0	2	10	1.833333333	0.066666667	0	0
2	2	7	6.53	0	2	30	0.9	0.183333333	0	0
2	2	8	8.56	0	2	2	5.383333333	NA	0	9.616666667
2	2	9	14.67	0	2	10	8.216666667	0.983333333	0	0
3	11	1	18.94	1	2	2	1.266666667	NA	0	13.73333333
3	11	2	10.18	0	2	10	0.4	0.25	0	0
3	11	3	7.84	0	2	2	5.016666667	0.866666667	0	0
3	11	4	16.61	0	2	4	0.466666667	1.333333333	0	0
3	11	5	8.88	0	2	30	0.466666667	0.266666667	0	0
3	11	6	7.54	0	2	10	0.933333333	3.233333333	0	0
3	11	7	6.53	0	2	30	0.45	0.066666667	0	0
3	11	8	8.56	0	2	2	NA	NA	15	0
3	11	9	14.67	0	2	10	5.05	2.216666667	0	0
4	24	1	18.94	0	2	2	17.63333333	1.25	0	0
4	24	2	10.18	0	2	10	0.033333333	0.8	0	0
4	24	3	7.84	0	2	2	1.616666667	1.266666667	0	0
4	24	4	16.61	0	2	4	1.366666667	4.25	0	0
4	24	5	8.88	0	2	30	0.6	0.316666667	0	0
4	24	6	7.54	0	2	10	0.116666667	0.066666667	0	0
4	24	7	6.53	0	2	30	0.066666667	0.6	0	0
4	24	8	8.56	0	2	2	9.716666667	NA	0	5.283333333
4	24	9	14.67	1	2	10	2.133333333	2.083333333	0	0
5	17	1	18.94	0	2	2	8.633333333	NA	0	6.366666667
5	17	2	10.18	0	2	10	1.116666667	0.45	0	0
5	17	3	7.84	0	2	2	1.733333333	NA	0	8.266666667
5	17	4	16.61	0	2	4	0.766666667	7.183333333	0	0
5	17	5	8.88	0	2	30	0.216666667	0.008333333	0	0
5	17	6	7.54	0	2	10	3.116666667	0.616666667	0	0
5	17	7	6.53	1	2	30	0.166666667	0.016666667	0	0
5	17	8	8.56	0	2	2	5.866666667	1.133333333	0	0
5	17	9	14.67	0	2	10	0.116666667	2	0	0
6	21	1	18.94	0	2	2	10.53333333	0.65	0	0
6	21	2	10.18	1	2	10	1.466666667	1.033333333	0	0
6	21	3	7.84	0	2	2	4.033333333	1.4	0	0
6	21	4	16.61	0	2	4	5.266666667	NA	0	9.733333333
6	21	5	8.88	0	2	30	0.233333333	0.966666667	0	0
6	21	6	7.54	0	2	10	0.266666667	1.233333333	0	0
6	21	7	6.53	0	2	30	2.05	0.566666667	0	0
6	21	8	8.56	0	2	2	1.966666667	NA	0	13.03333333
6	21	9	14.67	0	2	10	0.8	6.15	0	0
7	5	1	18.94	0	2	2	9.3	5.25	0	0
7	5	2	10.18	0	2	10	3.383333333	0.116666667	0	0
7	5	3	7.84	0	2	2	5.3	NA	0	9.7
7	5	4	16.61	0	2	4	9.25	NA	0	5.75
7	5	5	8.88	1	2	30	1.616666667	0.383333333	0	0
7	5	6	7.54	0	2	10	2.266666667	3.05	0	0
7	5	7	6.53	0	2	30	0.083333333	1.033333333	0	0
7	5	8	8.56	0	2	2	12.41666667	NA	0	2.583333333
7	5	9	14.67	0	2	10	3.366666667	0.916666667	0	0
8	8	1	18.94	0	2	2	1.216666667	NA	0	13.78333333
8	8	2	10.18	0	2	10	2.35	1.45	0	0
8	8	3	7.84	1	2	2	10.15	2.933333333	0	0
8	8	4	16.61	0	2	4	12.33333333	NA	0	2.666666667
8	8	5	8.88	0	2	30	2.9	0.516666667	0	0
8	8	6	7.54	0	2	10	4.3	3.45	0	0
8	8	7	6.53	0	2	30	0.583333333	0.5	0	0
8	8	8	8.56	0	2	2	11.01666667	NA	0	3.983333333
8	8	9	14.67	0	2	10	3.216666667	2.333333333	0	0
9	3	1	18.94	0	2	2	9.516666667	NA	0	5.483333333
9	3	2	10.18	0	2	10	1.316666667	2.516666667	0	0
9	3	3	7.84	0	2	2	2.583333333	1.466666667	0	0
9	3	4	16.61	0	2	4	10.18333333	NA	0	4.816666667
9	3	5	8.88	0	2	30	0.5	0.633333333	0	0
9	3	6	7.54	0	2	10	0.666666667	5.516666667	0	0
9	3	7	6.53	0	2	30	0.166666667	0.033333333	0	0
9	3	8	8.56	0	2	2	1.466666667	NA	0	13.53333333
9	3	9	14.67	1	2	10	0.966666667	2.266666667	0	0
10	3	1	18.94	0	2	2	3.983333333	NA	0	11.01666667
10	3	2	10.18	0	2	10	2.566666667	1.15	0	0
10	3	3	7.84	0	2	2	NA	NA	15.01	0
10	3	4	16.61	0	2	4	2.816666667	4.083333333	0	0
10	3	5	8.88	0	2	30	0.416666667	0.816666667	0	0
10	3	6	7.54	0	2	10	0.933333333	1.2	0	0
10	3	7	6.53	0	2	30	0.283333333	0.066666667	0	0
10	3	8	8.56	1	2	2	NA	NA	15	0
10	3	9	14.67	0	2	10	0.25	0.733333333	0	0
11	10	1	18.94	0	2	2	NA	NA	15	0
11	10	2	10.18	0	2	10	5.566666667	2.716666667	0	0
11	10	3	7.84	0	2	2	8.466666667	NA	0	6.533333333
11	10	4	16.61	0	2	4	NA	NA	15	0
11	10	5	8.88	0	2	30	0.483333333	2.6	0	0
11	10	6	7.54	0	2	10	0.65	5.483333333	0	0
11	10	7	6.53	1	2	30	0.816666667	0.783333333	0	0
11	10	8	8.56	0	2	2	5.516666667	9.416666667	0	0
11	10	9	14.67	0	2	10	2.633333333	2.233333333	0	0
12	16	1	18.94	0	2	2	NA	NA	15	0
12	16	2	10.18	1	2	10	3.333333333	4.833333333	0	0
12	16	3	7.84	0	2	2	0.1	5.866666667	0	0
12	16	4	16.61	0	2	4	7.283333333	4.783333333	0	0
12	16	5	8.88	0	2	30	0.583333333	0.383333333	0	0
12	16	6	7.54	0	2	10	2.45	0.133333333	0	0
12	16	7	6.53	0	2	30	0.966666667	0.216666667	0	0
12	16	8	8.56	0	2	2	NA	NA	15	0
12	16	9	14.67	0	2	10	6.933333333	0.4	0	0
13	30	1	18.94	0	2	2	11.28333333	NA	0	3.716666667
13	30	2	10.18	0	2	10	2.45	3.216666667	0	0
13	30	3	7.84	0	2	2	1.366666667	3.983333333	0	0
13	30	4	16.61	0	2	4	2.216666667	9.916666667	0	0
13	30	5	8.88	1	2	30	0.4	0.933333333	0	0
13	30	6	7.54	0	2	10	0.633333333	5.083333333	0	0
13	30	7	6.53	0	2	30	0.066666667	1.2	0	0
13	30	8	8.56	0	2	2	9.966666667	NA	0	5.033333333
13	30	9	14.67	0	2	10	1.833333333	2.316666667	0	0
14	10	1	18.94	0	2	2	0.066666667	NA	0	14.93333333
14	10	2	10.18	0	2	10	1.716666667	1.5	0	0
14	10	3	7.84	1	2	2	2.233333333	1.033333333	0	0
14	10	4	16.61	0	2	4	4.783333333	NA	0	10.21666667
14	10	5	8.88	0	2	30	0.65	0.166666667	0	0
14	10	6	7.54	0	2	10	0.45	0.833333333	0	0
14	10	7	6.53	0	2	30	0.05	0.016666667	0	0
14	10	8	8.56	0	2	2	7	NA	0	8
14	10	9	14.67	0	2	10	1.416666667	1.616666667	0	0
1	4	1	18.94	0	3	2	3.333333333	8.933333333	0	0
1	4	2	10.18	0	3	30	1.25	1.383333333	0	0
1	4	3	7.84	0	3	4	2.516666667	0.733333333	0	0
1	4	4	16.61	0	3	10	3.816666667	NA	0	11.18333333
1	4	5	8.88	0	3	4	4.75	2	0	0
1	4	6	7.54	1	3	10	3.216666667	2.233333333	0	0
1	4	7	6.53	0	3	10	2.4	2.883333333	0	0
1	4	8	8.56	0	3	4	4.65	0.183333333	0	0
1	4	9	14.67	0	3	30	0.2	0.55	0	0
2	2	1	18.94	0	3	2	6.083333333	NA	0	8.916666667
2	2	2	10.18	0	3	30	1.033333333	1.333333333	0	0
2	2	3	7.84	1	3	4	1.966666667	0.45	0	0
2	2	4	16.61	0	3	10	1.666666667	5.833333333	0	0
2	2	5	8.88	0	3	4	4.75	0.25	0	0
2	2	6	7.54	0	3	10	0.433333333	0.033333333	0	0
2	2	7	6.53	0	3	10	3.883333333	1.1	0	0
2	2	8	8.56	0	3	4	1.583333333	4.65	0	0
2	2	9	14.67	0	3	30	0.4	0.65	0	0
3	11	1	18.94	1	3	2	6.983333333	NA	0	8.016666667
3	11	2	10.18	0	3	30	1.233333333	1.633333333	0	0
3	11	3	7.84	0	3	4	0.666666667	1.316666667	0	0
3	11	4	16.61	0	3	10	1.866666667	0.316666667	0	0
3	11	5	8.88	0	3	4	6	0.483333333	0	0
3	11	6	7.54	0	3	10	1.15	1.316666667	0	0
3	11	7	6.53	0	3	10	1.083333333	0.416666667	0	0
3	11	8	8.56	0	3	4	0.7	2.716666667	0	0
3	11	9	14.67	0	3	30	0.533333333	0.783333333	0	0
4	24	1	18.94	0	3	2	5.583333333	1.233333333	0	0
4	24	2	10.18	0	3	30	1.333333333	0.133333333	0	0
4	24	3	7.84	0	3	4	1.033333333	0.533333333	0	0
4	24	4	16.61	0	3	10	0.966666667	5.366666667	0	0
4	24	5	8.88	0	3	4	2.483333333	1.016666667	0	0
4	24	6	7.54	0	3	10	1.166666667	0.55	0	0
4	24	7	6.53	0	3	10	2.75	1.816666667	0	0
4	24	8	8.56	0	3	4	0.966666667	7.666666667	0	0
4	24	9	14.67	1	3	30	0.55	0.516666667	0	0
5	17	1	18.94	0	3	2	3.566666667	4.383333333	0	0
5	17	2	10.18	0	3	30	0.05	0.083333333	0	0
5	17	3	7.84	0	3	4	1.2	1.933333333	0	0
5	17	4	16.61	0	3	10	0.333333333	0.333333333	0	0
5	17	5	8.88	0	3	4	10.5	NA	0	4.5
5	17	6	7.54	0	3	10	0.583333333	0.183333333	0	0
5	17	7	6.53	1	3	10	0.483333333	0.066666667	0	0
5	17	8	8.56	0	3	4	0.316666667	3.15	0	0
5	17	9	14.67	0	3	30	0.05	0.016666667	0	0
6	21	1	18.94	0	3	2	3.366666667	NA	0	11.63333333
6	21	2	10.18	1	3	30	0.133333333	0.15	0	0
6	21	3	7.84	0	3	4	0.3	2.85	0	0
6	21	4	16.61	0	3	10	0.333333333	2.316666667	0	0
6	21	5	8.88	0	3	4	5.5	0.65	0	0
6	21	6	7.54	0	3	10	0.566666667	1.15	0	0
6	21	7	6.53	0	3	10	4.583333333	3.666666667	0	0
6	21	8	8.56	0	3	4	0.266666667	0.116666667	0	0
6	21	9	14.67	0	3	30	0.216666667	1.183333333	0	0
7	5	1	18.94	0	3	2	1.683333333	NA	0	13.31666667
7	5	2	10.18	0	3	30	0.716666667	2.116666667	0	0
7	5	3	7.84	0	3	4	0.066666667	3.166666667	0	0
7	5	4	16.61	0	3	10	0.7	0.25	0	0
7	5	5	8.88	1	3	4	1.466666667	5.966666667	0	0
7	5	6	7.54	0	3	10	0.3	1.433333333	0	0
7	5	7	6.53	0	3	10	0.2	2.283333333	0	0
7	5	8	8.56	0	3	4	9.166666667	0.483333333	0	0
7	5	9	14.67	0	3	30	0.116666667	0.25	0	0
8	8	1	18.94	0	3	2	5.25	NA	0	9.75
8	8	2	10.18	0	3	30	1.183333333	0.283333333	0	0
8	8	3	7.84	1	3	4	14.93333333	NA	0	0.066666667
8	8	4	16.61	0	3	10	5.55	1.483333333	0	0
8	8	5	8.88	0	3	4	11	NA	0	4
8	8	6	7.54	0	3	10	0.316666667	4.216666667	0	0
8	8	7	6.53	0	3	10	10.4	0.35	0	0
8	8	8	8.56	0	3	4	12.5	NA	0	2.5
8	8	9	14.67	0	3	30	1.916666667	0.933333333	0	0
9	3	1	18.94	0	3	2	5.733333333	6.633333333	0	0
9	3	2	10.18	0	3	30	0.983333333	3.45	0	0
9	3	3	7.84	0	3	4	1.35	4.666666667	0	0
9	3	4	16.61	0	3	10	4.516666667	2.466666667	0	0
9	3	5	8.88	0	3	4	NA	NA	15	0
9	3	6	7.54	0	3	10	2.4	0.1	0	0
9	3	7	6.53	0	3	10	5.2	2.266666667	0	0
9	3	8	8.56	0	3	4	0.5	1.3	0	0
9	3	9	14.67	1	3	30	0.433333333	0.533333333	0	0
10	3	1	18.94	0	3	2	13.08333333	NA	0	1.916666667
10	3	2	10.18	0	3	30	0.1	2.366666667	0	0
10	3	3	7.84	0	3	4	4.233333333	0.716666667	0	0
10	3	4	16.61	0	3	10	2.15	0.45	0	0
10	3	5	8.88	0	3	4	12.41666667	1.466666667	0	0
10	3	6	7.54	0	3	10	0.783333333	2.3	0	0
10	3	7	6.53	0	3	10	5.933333333	2.15	0	0
10	3	8	8.56	1	3	4	1.8	8.033333333	0	0
10	3	9	14.67	0	3	30	1.4	0.583333333	0	0
11	10	1	18.94	0	3	2	1.466666667	1.733333333	0	0
11	10	2	10.18	0	3	30	4.766666667	1.466666667	0	0
11	10	3	7.84	0	3	4	0.983333333	3.8	0	0
11	10	4	16.61	0	3	10	0.116666667	10.3	0	0
11	10	5	8.88	0	3	4	5.483333333	0.883333333	0	0
11	10	6	7.54	0	3	10	0.016666667	2.366666667	0	0
11	10	7	6.53	1	3	10	9	0.95	0	0
11	10	8	8.56	0	3	4	4.016666667	NA	0	10.98333333
11	10	9	14.67	0	3	30	0.483333333	0.183333333	0	0
12	16	1	18.94	0	3	2	2.333333333	7.666666667	0	0
12	16	2	10.18	1	3	30	2.733333333	1	0	0
12	16	3	7.84	0	3	4	4.25	6.5	0	0
12	16	4	16.61	0	3	10	1.683333333	1.85	0	0
12	16	5	8.88	0	3	4	10.61666667	NA	0	4.383333333
12	16	6	7.54	0	3	10	1.2	0.166666667	0	0
12	16	7	6.53	0	3	10	2.466666667	2.4	0	0
12	16	8	8.56	0	3	4	6.866666667	NA	0	8.133333333
12	16	9	14.67	0	3	30	2.216666667	2.316666667	0	0
13	30	1	18.94	0	3	2	NA	NA	15	0
13	30	2	10.18	0	3	30	1.166666667	1.633333333	0	0
13	30	3	7.84	0	3	4	3.4	3.016666667	0	0
13	30	4	16.61	0	3	10	0.116666667	0.416666667	0	0
13	30	5	8.88	1	3	4	3.6	NA	0	11.4
13	30	6	7.54	0	3	10	0.983333333	0.616666667	0	0
13	30	7	6.53	0	3	10	7	2.05	0	0
13	30	8	8.56	0	3	4	2.216666667	5.15	0	0
13	30	9	14.67	0	3	30	0.566666667	0.683333333	0	0
14	10	1	18.94	0	3	2	NA	NA	15	0
14	10	2	10.18	0	3	30	1.183333333	0.666666667	0	0
14	10	3	7.84	1	3	4	NA	NA	15	0
14	10	4	16.61	0	3	10	0.2	0.7	0	0
14	10	5	8.88	0	3	4	8.816666667	NA	0	6.183333333
14	10	6	7.54	0	3	10	0.266666667	0.816666667	0	0
14	10	7	6.53	0	3	10	0.183333333	0.2	0	0
14	10	8	8.56	0	3	4	0.083333333	2.816666667	0	0
14	10	9	14.67	0	3	30	0.45	0.283333333	0	0
1	4	1	18.94	0	4	30	4.05	0.366666667	0	0
1	4	2	10.18	0	4	4	11.56666667	NA	0	3.433333333
1	4	3	7.84	0	4	10	5.483333333	3.366666667	0	0
1	4	4	16.61	0	4	30	1.216666667	2.483333333	0	0
1	4	5	8.88	0	4	10	4.716666667	6.3	0	0
1	4	6	7.54	1	4	4	NA	NA	15	0
1	4	7	6.53	0	4	4	NA	NA	15	0
1	4	8	8.56	0	4	4	NA	NA	15	0
1	4	9	14.67	0	4	10	12.58333333	0.45	0	0
2	2	1	18.94	0	4	30	1.35	1.85	0	0
2	2	2	10.18	0	4	4	NA	NA	15	0
2	2	3	7.84	1	4	10	1.6	3.283333333	0	0
2	2	4	16.61	0	4	30	2.516666667	0.8	0	0
2	2	5	8.88	0	4	10	2.783333333	4.533333333	0	0
2	2	6	7.54	0	4	4	3.633333333	NA	0	11.36666667
2	2	7	6.53	0	4	4	NA	NA	15	0
2	2	8	8.56	0	4	4	NA	NA	15	0
2	2	9	14.67	0	4	10	1.866666667	2.2	0	0
3	11	1	18.94	1	4	30	1.433333333	0.45	0	0
3	11	2	10.18	0	4	4	2.266666667	11.01666667	0	0
3	11	3	7.84	0	4	10	1.016666667	3.316666667	0	0
3	11	4	16.61	0	4	30	0.65	0.033333333	0	0
3	11	5	8.88	0	4	10	2.016666667	0.516666667	0	0
3	11	6	7.54	0	4	4	2.85	1.083333333	0	0
3	11	7	6.53	0	4	4	6.916666667	NA	0	8.083333333
3	11	8	8.56	0	4	4	1.55	4.566666667	0	0
3	11	9	14.67	0	4	10	11.66666667	1.816666667	0	0
4	24	1	18.94	0	4	30	5.033333333	1	0	0
4	24	2	10.18	0	4	4	NA	NA	15	0
4	24	3	7.84	0	4	10	3.766666667	2.433333333	0	0
4	24	4	16.61	0	4	30	2.233333333	2.166666667	0	0
4	24	5	8.88	0	4	10	3.466666667	6.716666667	0	0
4	24	6	7.54	0	4	4	NA	NA	15	0
4	24	7	6.53	0	4	4	13.21666667	NA	0	1.783333333
4	24	8	8.56	0	4	4	3.7	NA	0	11.3
4	24	9	14.67	1	4	10	0.566666667	4.366666667	0	0
5	17	1	18.94	0	4	30	0.683333333	1.4	0	0
5	17	2	10.18	0	4	4	0.466666667	0.016666667	0	0
5	17	3	7.84	0	4	10	0.75	1.916666667	0	0
5	17	4	16.61	0	4	30	0.033333333	0.133333333	0	0
5	17	5	8.88	0	4	10	4.65	0.516666667	0	0
5	17	6	7.54	0	4	4	7	0.5	0	0
5	17	7	6.53	1	4	4	10.45	NA	0	3.55
5	17	8	8.56	0	4	4	5.45	1.483333333	0	0
5	17	9	14.67	0	4	10	5.583333333	4.733333333	0	0
6	21	1	18.94	0	4	30	0.966666667	0.166666667	0	0
6	21	2	10.18	1	4	4	0.95	9.6	0	0
6	21	3	7.84	0	4	10	1.416666667	0.9	0	0
6	21	4	16.61	0	4	30	1.966666667	1.283333333	0	0
6	21	5	8.88	0	4	10	13.25	0.716666667	0	0
6	21	6	7.54	0	4	4	3.816666667	NA	0	11.18333333
6	21	7	6.53	0	4	4	11.91666667	NA	0	3.083333333
6	21	8	8.56	0	4	4	5.15	8.35	0	0
6	21	9	14.67	0	4	10	1.766666667	1.366666667	0	0
7	5	1	18.94	0	4	30	2.566666667	0.433333333	0	0
7	5	2	10.18	0	4	4	6.1	NA	0	8.9
7	5	3	7.84	0	4	10	5.783333333	2.166666667	0	0
7	5	4	16.61	0	4	30	0.533333333	0.533333333	0	0
7	5	5	8.88	1	4	10	10.1	0.233333333	0	0
7	5	6	7.54	0	4	4	NA	NA	15	0
7	5	7	6.53	0	4	4	NA	NA	15	0
7	5	8	8.56	0	4	4	8.8	3.183333333	0	0
7	5	9	14.67	0	4	10	10.41666667	4	0	0
8	8	1	18.94	0	4	30	10.7	1.933333333	0	0
8	8	2	10.18	0	4	4	NA	NA	13.2	0
8	8	3	7.84	1	4	10	6.466666667	4.7	0	0
8	8	4	16.61	0	4	30	13.18333333	NA	0	1.816666667
8	8	5	8.88	0	4	10	7.383333333	3.75	0	0
8	8	6	7.54	0	4	4	NA	NA	15	0
8	8	7	6.53	0	4	4	NA	NA	15	0
8	8	8	8.56	0	4	4	NA	NA	15	0
8	8	9	14.67	0	4	10	0.65	NA	0	14.35
9	3	1	18.94	0	4	30	0.516666667	8.95	0	0
9	3	2	10.18	0	4	4	9.133333333	NA	0	5.866666667
9	3	3	7.84	0	4	10	5.35	1.016666667	0	0
9	3	4	16.61	0	4	30	0.9	1.5	0	0
9	3	5	8.88	0	4	10	2.333333333	4	0	0
9	3	6	7.54	0	4	4	NA	NA	15	0
9	3	7	6.53	0	4	4	11.13333333	NA	0	3.866666667
9	3	8	8.56	0	4	4	NA	NA	15	0
9	3	9	14.67	1	4	10	NA	NA	15	0
10	3	1	18.94	0	4	30	7.416666667	NA	0	7.583333333
10	3	2	10.18	0	4	4	2.65	NA	0	12.35
10	3	3	7.84	0	4	10	8.666666667	1.383333333	0	0
10	3	4	16.61	0	4	30	0.6	7.083333333	0	0
10	3	5	8.88	0	4	10	9.75	NA	0	5.28
10	3	6	7.54	0	4	4	NA	NA	15	0
10	3	7	6.53	0	4	4	NA	NA	15	0
10	3	8	8.56	1	4	4	NA	NA	15	0
10	3	9	14.67	0	4	10	8.216666667	1.216666667	0	0
11	10	1	18.94	0	4	30	10.7	NA	0	4.3
11	10	2	10.18	0	4	4	9.15	0.983333333	0	0
11	10	3	7.84	0	4	10	0.716666667	3.083333333	0	0
11	10	4	16.61	0	4	30	1.4	0.066666667	0	0
11	10	5	8.88	0	4	10	3.5	9.983333333	0	0
11	10	6	7.54	0	4	4	NA	NA	15	0
11	10	7	6.53	1	4	4	NA	NA	15	0
11	10	8	8.56	0	4	4	NA	NA	15	0
11	10	9	14.67	0	4	10	NA	NA	15	0
12	16	1	18.94	0	4	30	12.61666667	1.166666667	0	0
12	16	2	10.18	1	4	4	9.733333333	0.233333333	0	0
12	16	3	7.84	0	4	10	14.78333333	NA	0	0.216666667
12	16	4	16.61	0	4	30	0.883333333	0.316666667	0	0
12	16	5	8.88	0	4	10	5	1.133333333	0	0
12	16	6	7.54	0	4	4	NA	NA	15	0
12	16	7	6.53	0	4	4	8.85	NA	0	6.15
12	16	8	8.56	0	4	4	NA	NA	15	0
12	16	9	14.67	0	4	10	NA	NA	15	0
13	30	1	18.94	0	4	30	8.083333333	1.116666667	0	0
13	30	2	10.18	0	4	4	3.466666667	4.566666667	0	0
13	30	3	7.84	0	4	10	0.633333333	5.183333333	0	0
13	30	4	16.61	0	4	30	2.55	1.6	0	0
13	30	5	8.88	1	4	10	6.533333333	7.8	0	0
13	30	6	7.54	0	4	4	9.6	NA	0	5.4
13	30	7	6.53	0	4	4	NA	NA	15	0
13	30	8	8.56	0	4	4	6.816666667	3.6	0	0
13	30	9	14.67	0	4	10	1.333333333	0.25	0	0
14	10	1	18.94	0	4	30	0.816666667	3.783333333	0	0
14	10	2	10.18	0	4	4	NA	NA	15	0
14	10	3	7.84	1	4	10	0.816666667	0.95	0	0
14	10	4	16.61	0	4	30	1.55	2.283333333	0	0
14	10	5	8.88	0	4	10	4.366666667	5.35	0	0
14	10	6	7.54	0	4	4	9.666666667	NA	0	5.333333333
14	10	7	6.53	0	4	4	NA	NA	15	0
14	10	8	8.56	0	4	4	NA	NA	15	0
14	10	9	14.67	0	4	10	2.433333333	0.283333333	0	0
1	4	1	18.94	0	5	30	NA	NA	15	0
1	4	2	10.18	0	5	4	NA	NA	15	0
1	4	3	7.84	0	5	4	NA	NA	15	0
1	4	4	16.61	0	5	10	3.016666667	NA	0	11.98333333
1	4	5	8.88	0	5	10	11.86666667	1.316666667	0	0
1	4	6	7.54	1	5	4	9.35	NA	0	5.65
1	4	7	6.53	0	5	30	1	3.333333333	0	0
1	4	8	8.56	0	5	10	NA	NA	15	0
1	4	9	14.67	0	5	4	NA	NA	15	0
2	2	1	18.94	0	5	30	5.933333333	2.433333333	0	0
2	2	2	10.18	0	5	4	NA	NA	15	0
2	2	3	7.84	1	5	4	NA	NA	15	0
2	2	4	16.61	0	5	10	2.716666667	3.683333333	0	0
2	2	5	8.88	0	5	10	8.066666667	0.866666667	0	0
2	2	6	7.54	0	5	4	0.116666667	NA	0	14.88333333
2	2	7	6.53	0	5	30	0.7	1.7	0	0
2	2	8	8.56	0	5	10	NA	NA	15	0
2	2	9	14.67	0	5	4	0.516666667	12.28333333	0	0
3	11	1	18.94	1	5	30	5.316666667	1.966666667	0	0
3	11	2	10.18	0	5	4	1.65	NA	0	13.35
3	11	3	7.84	0	5	4	7.166666667	1.683333333	0	0
3	11	4	16.61	0	5	10	0.883333333	12.66666667	0	0
3	11	5	8.88	0	5	10	2.066666667	0.583333333	0	0
3	11	6	7.54	0	5	4	2.916666667	5.6	0	0
3	11	7	6.53	0	5	30	0.7	0.816666667	0	0
3	11	8	8.56	0	5	10	1.7	0.3	0	0
3	11	9	14.67	0	5	4	6.683333333	NA	0	8.316666667
4	24	1	18.94	0	5	30	6.466666667	10.78333333	0	0
4	24	2	10.18	0	5	4	11.76666667	1.483333333	0	0
4	24	3	7.84	0	5	4	1.083333333	NA	0	13.91666667
4	24	4	16.61	0	5	10	NA	NA	7	0
4	24	5	8.88	0	5	10	8.533333333	NA	0	6.466666667
4	24	6	7.54	0	5	4	NA	NA	15	0
4	24	7	6.53	0	5	30	1.133333333	1.833333333	0	0
4	24	8	8.56	0	5	10	NA	NA	15	0
4	24	9	14.67	1	5	4	NA	NA	5	0
5	17	1	18.94	0	5	30	0.383333333	0.35	0	0
5	17	2	10.18	0	5	4	0.85	0.583333333	0	0
5	17	3	7.84	0	5	4	1.15	0.033333333	0	0
5	17	4	16.61	0	5	10	5.166666667	8.583333333	0	0
5	17	5	8.88	0	5	10	3.533333333	0.85	0	0
5	17	6	7.54	0	5	4	0.85	6.066666667	0	0
5	17	7	6.53	1	5	30	3.816666667	0.133333333	0	0
5	17	8	8.56	0	5	10	3.966666667	0.966666667	0	0
5	17	9	14.67	0	5	4	12.88333333	NA	0	2.116666667
6	21	1	18.94	0	5	30	5.383333333	1.516666667	0	0
6	21	2	10.18	1	5	4	NA	NA	15	0
6	21	3	7.84	0	5	4	14.91666667	NA	0	0.083333333
6	21	4	16.61	0	5	10	NA	NA	15	0
6	21	5	8.88	0	5	10	6.666666667	NA	0	8.333333333
6	21	6	7.54	0	5	4	3.9	NA	0	11.1
6	21	7	6.53	0	5	30	0.5	1.466666667	0	0
6	21	8	8.56	0	5	10	1.45	NA	0	13.55
6	21	9	14.67	0	5	4	NA	NA	15	0
7	5	1	18.94	0	5	30	11.25	NA	0	3.75
7	5	2	10.18	0	5	4	11.28333333	NA	0	3.716666667
7	5	3	7.84	0	5	4	NA	NA	15	0
7	5	4	16.61	0	5	10	NA	NA	15	0
7	5	5	8.88	1	5	10	7.333333333	6.45	0	0
7	5	6	7.54	0	5	4	NA	NA	15	0
7	5	7	6.53	0	5	30	2.033333333	2.083333333	0	0
7	5	8	8.56	0	5	10	9.166666667	NA	0	5.833333333
7	5	9	14.67	0	5	4	NA	NA	15	0
8	8	1	18.94	0	5	30	13.53333333	NA	0	1.466666667
8	8	2	10.18	0	5	4	NA	NA	13.2	0
8	8	3	7.84	1	5	4	NA	NA	15	0
8	8	4	16.61	0	5	10	6.35	7.316666667	0	0
8	8	5	8.88	0	5	10	NA	NA	15	0
8	8	6	7.54	0	5	4	NA	NA	15	0
8	8	7	6.53	0	5	30	11.5	NA	0	3.5
8	8	8	8.56	0	5	10	NA	NA	15	0
8	8	9	14.67	0	5	4	NA	NA	15	0
9	3	1	18.94	0	5	30	1.25	NA	0	13.75
9	3	2	10.18	0	5	4	NA	NA	15	0
9	3	3	7.84	0	5	4	5.883333333	0.133333333	0	0
9	3	4	16.61	0	5	10	0.25	5.916666667	0	0
9	3	5	8.88	0	5	10	NA	NA	15	0
9	3	6	7.54	0	5	4	NA	NA	15	0
9	3	7	6.53	0	5	30	7.583333333	3.45	0	0
9	3	8	8.56	0	5	10	NA	NA	15	0
9	3	9	14.67	1	5	4	NA	NA	15	0
10	3	1	18.94	0	5	30	2.333333333	10.6	0	0
10	3	2	10.18	0	5	4	NA	NA	15	0
10	3	3	7.84	0	5	4	NA	NA	15.01	0
10	3	4	16.61	0	5	10	NA	NA	15.01	0
10	3	5	8.88	0	5	10	14.61666667	NA	0	0.413333333
10	3	6	7.54	0	5	4	NA	NA	15	0
10	3	7	6.53	0	5	30	0.966666667	2.533333333	0	0
10	3	8	8.56	1	5	10	1.583333333	NA	0	13.41666667
10	3	9	14.67	0	5	4	NA	NA	15	0
11	10	1	18.94	0	5	30	1.783333333	NA	0	13.21666667
11	10	2	10.18	0	5	4	NA	NA	15	0
11	10	3	7.84	0	5	4	NA	NA	15	0
11	10	4	16.61	0	5	10	NA	NA	15	0
11	10	5	8.88	0	5	10	NA	NA	15	0
11	10	6	7.54	0	5	4	NA	NA	15	0
11	10	7	6.53	1	5	30	4.983333333	3.083333333	0	0
11	10	8	8.56	0	5	10	2.066666667	NA	0	12.93333333
11	10	9	14.67	0	5	4	NA	NA	15	0
12	16	1	18.94	0	5	30	12.98333333	NA	0	2.016666667
12	16	2	10.18	1	5	4	10.95	NA	0	4.05
12	16	3	7.84	0	5	4	NA	NA	15	0
12	16	4	16.61	0	5	10	8.566666667	NA	0	6.433333333
12	16	5	8.88	0	5	10	3.5	0.233333333	0	0
12	16	6	7.54	0	5	4	NA	NA	15	0
12	16	7	6.53	0	5	30	1.666666667	2.716666667	0	0
12	16	8	8.56	0	5	10	6.6	5.633333333	0	0
12	16	9	14.67	0	5	4	NA	NA	15	0
13	30	1	18.94	0	5	30	0.183333333	7.15	0	0
13	30	2	10.18	0	5	4	NA	NA	15	0
13	30	3	7.84	0	5	4	NA	NA	15	0
13	30	4	16.61	0	5	10	5.9	NA	0	9.1
13	30	5	8.88	1	5	10	3.433333333	6.333333333	0	0
13	30	6	7.54	0	5	4	NA	NA	15	0
13	30	7	6.53	0	5	30	5.866666667	0.533333333	0	0
13	30	8	8.56	0	5	10	5.7	5.066666667	0	0
13	30	9	14.67	0	5	4	NA	NA	15	0
14	10	1	18.94	0	5	30	5.583333333	0.95	0	0
14	10	2	10.18	0	5	4	10.98333333	NA	0	4.016666667
14	10	3	7.84	1	5	4	NA	NA	15	0
14	10	4	16.61	0	5	10	11.23333333	3.516666667	0	0
14	10	5	8.88	0	5	10	3.566666667	5.366666667	0	0
14	10	6	7.54	0	5	4	3.583333333	1.366666667	0	0
14	10	7	6.53	0	5	30	1.083333333	0.2	0	0
14	10	8	8.56	0	5	10	1.35	2.816666667	0	0
14	10	9	14.67	0	5	4	NA	NA	15	0
END										
